# Identifying Drug‐Induced Metabolic Signatures in Comparative Untargeted NMR Metabolomics Using Difference Spectra

**DOI:** 10.1155/bca/9636071

**Published:** 2026-10-23

**Authors:** Giorgio Di Paco, Veronica Ghini, Michele Mannelli, Tania Gamberi, Antonio Rosato, Luigi Messori, Paola Turano

**Affiliations:** ^1^ Department of Chemistry “Ugo Schiff” University of Florence Florence 50019 Italy unifi.it; ^2^ Department of Experimental and Clinical Biomedical Sciences “Mario Serio” University of Florence Florence 50134 Italy unifi.it; ^3^ Magnetic Resonance Center (CERM) University of Florence Sesto Fiorentino 50019 Italy unifi.it

**Keywords:** ^1^H NMR, cell line metabolomics, difference spectra, drug-induced metabolic signatures, untargeted metabolomics

## Abstract

Untargeted NMR‐based metabolomics is widely applied to investigate drug‐induced metabolic alterations. However, comparative analyses across different biological models are often limited by dominant baseline variability arising from intrinsic metabolic differences and distinct culture conditions. This variability may obscure treatment‐related signatures and compromise both multivariate and univariate statistical analyses. Here, we propose a data‐processing strategy based on difference spectra as input for statistical modeling. For each treatment condition, difference spectra are first calculated by subtracting each paired control binned spectrum from its corresponding treated binned spectrum. These replicate‐level binned difference spectra are then averaged to reduce intersample variability while enhancing the detection of treatment‐induced metabolic changes within an untargeted framework. The strategy was validated by testing four structurally diverse gold(I) compounds in two ovarian cancer cell lines characterized by significant differences in basal metabolism and grown in distinct media. While conventional multivariate analysis of individual spectra failed to extract shared drug‐related signatures, the difference‐spectra approach enabled the identification of conserved metabolic perturbations. Altered pathways across cell models were then evaluated based on univariate analysis. Overall, the proposed method provides a robust and broadly applicable analytical tool for comparative untargeted metabolomics, facilitating the identification of common metabolic fingerprints despite substantial baseline heterogeneity.

## 1. Introduction

NMR‐based metabolomics is a powerful analytical technique for investigating the effects of drugs or drug candidates [1–3]. In particular, preclinical drug testing and the study of cellular processes rely on in vitro models [4–6], which involve profiling metabolic changes in cell cultures through the combined analysis of cell lysates (endometabolome) and corresponding growth media (GM) (exometabolome [7]). Although generally less sensitive than mass spectrometry, one‐dimensional (1D) ^1^H NMR is particularly well‐suited for screening applications [8–12]. It provides reliable and quantitative data with minimal sample preparation and rapid acquisition times. This enables comparative analysis of multiple experimental conditions. Multivariate and univariate statistical analyses are typically employed to highlight spectral differences between control and treated groups or among different treatments (e.g., exposure time or drug type), while pathway analysis maps significantly altered metabolites onto metabolic networks, elucidating drug‐induced cellular responses [13].

Metallodrugs and metal‐based complexes developed as anticancer candidates act by targeting DNA or intracellular proteins [14–16]. In this context, NMR‐based metabolomics has proven to be a powerful tool for elucidating the mechanisms of action of platinum‐containing anticancer agents, including cisplatin and structurally related complexes, by revealing treatment‐induced perturbations of intracellular metabolic pathways [17–24]. More recently, the same metabolomic approach has been extended to the investigation of gold‐based anticancer agents, a rapidly expanding class of metallodrugs with mechanisms of action that differ substantially from those of platinum compounds. Gold compounds have emerged as a promising class of anticancer agents, showing significant in vitro and in vivo activity [25–28]. In previous studies, we performed extensive mechanistic investigations on several gold(I) compounds exhibiting marked cytotoxic activity against A2780 human ovarian cancer cells, both cisplatin‐sensitive and resistant [29–36]. Four structurally diverse gold (I) compounds were selected for detailed study: auranofin (AF), aurothiomalate (AuTM), Au(NHC)Cl (CN1), and Au(NHC)_2_PF_6_ (CN2), Scheme 1. These compounds induced distinct metabolic alterations [32]. AF was found to markedly increase glutathione levels in response to oxidative stress [29], whereas AuTM caused significant dysregulation of the tricarboxylic acid (TCA) cycle [31]. The two gold–carbene complexes shifted cellular metabolism toward a more glycolytic phenotype, with increased lactate production, suggesting a common mode of action, albeit with different intensities [30]. These findings were corroborated by independent proteomic and biochemical analyses [34–36].

**SCHEME 1:**
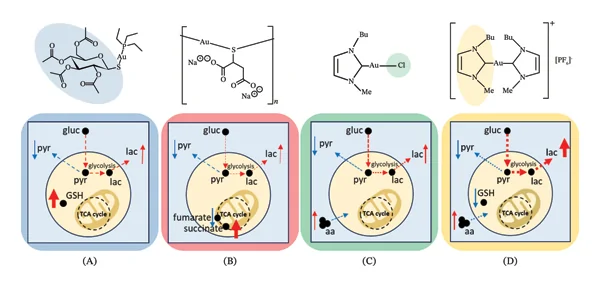
The four selected gold(I) compounds and their effects in A2780 human ovarian cancer cells. (A) Auranofin (AF), (B) aurothiomalate (AuTM), (C) Au(NHC)Cl (CN1), and (D) Au(NHC)_2_PF_6_ (CN2). Top: chemical structures, with leaving groups highlighted for AF, CN1, and CN2. Bottom: schematic representation of the main metabolic pathways identified as significantly altered upon treatment. Dashed arrows indicate the direction of metabolic fluxes; solid lines indicate changes in metabolite concentrations. Arrow thickness reflects the magnitude of the change. Red = upregulation/increase, blue = downregulation/decrease.

Although these studies demonstrated that NMR metabolomics can effectively characterize the metabolic response to structurally diverse gold(I) compounds, they were all performed in a single cellular model. A2780 and SKOV‐3 represent biologically distinct ovarian cancer cell models that differ in genetic background, metabolic phenotype, and drug sensitivity [37]. Evaluating drug‐induced metabolic responses in both models therefore provides a stringent test for distinguishing metabolic perturbations that are conserved across ovarian cancer cell lines and thus more likely to reflect compound‐specific mechanisms of action from those that are cell line‐dependent. More generally, previous metabolomic investigations of metallodrugs have rarely addressed whether drug‐induced metabolic signatures are conserved across biologically distinct cellular systems or are instead dominated by cell line‐specific metabolic features. The latter represents a major challenge for comparative metabolomics, as it hampers the identification of metabolic signatures that genuinely reflect shared drug action rather than the intrinsic characteristics of the cellular model. Consequently, analytical strategies capable of attenuating baseline metabolic differences while preserving treatment‐induced responses are highly desirable.

In comparative metabolomics, this approach may be affected by substantial confounding factors. Differences in basal metabolism, as well as the need for distinct culture media to ensure optimal growth, may dominate the spectral variance and obscure treatment‐related effects. This represents a general analytical challenge in untargeted metabolomics, where baseline variability can mask biologically relevant signatures.

To address this issue, we applied the same four gold(I) compounds to the SKOV‐3 ovarian cancer cell line. Even in the absence of treatment, SKOV‐3 cells displayed marked metabolic differences compared to the previously characterized A2780 cells. A2780 cells were cultured in RPMI 1640, whereas DMEM was found to be the optimal medium for SKOV‐3 cells. Beyond evident differences in the exometabolome, significant variations were also observed in the endometabolome, reflecting intrinsic basal metabolic disparities and potentially differential uptake from the culture medium, two factors that cannot be readily disentangled. Under these conditions, conventional multivariate analysis failed to extract a common drug‐related fingerprint across the two cell lines. Moreover, when each cell line is analyzed independently, similarities and differences in drug‐induced metabolic responses are typically evaluated by comparison of independently analyzed datasets, which makes it more challenging to distinguish conserved drug‐related signatures from those primarily reflecting the distinct biological backgrounds of the two models.

To overcome these limitations, we developed a strategy based on the use of difference spectra rather than individual spectra as the input for statistical analysis, thereby emphasizing treatment‐induced metabolic variations before multivariate analysis while minimizing contributions from basal metabolic differences. For each treatment in each cell line, difference spectra are first calculated by subtracting each paired control binned spectrum from its corresponding treated binned spectrum. The average binned difference spectra are then obtained as the mean of the corresponding replicate binned difference spectra. This generates profiles that emphasize treatment‐induced variations. By leveraging interspectral differences within an untargeted framework, this approach enabled the identification of drug‐induced effects shared across biologically distinct cell models directly at the multivariate analysis stage. This strategy enabled the identification of the metabolites and pathways most consistently affected by drug treatment, revealing significant similarities in the major metabolic responses of A2780 and SKOV‐3 cells for all four gold(I) compounds.

More broadly, this study demonstrates that the proposed strategy represents a reliable analytical tool for identifying common metabolic fingerprints of candidate drugs despite substantial basal metabolic differences between biological models, providing a robust basis for comparative untargeted metabolomic investigations in heterogeneous systems.

## 2. Methods

### 2.1. Cell Lines and Culture Conditions

Cell culture media, solutions, and plasticware were purchased from Euroclone (Milan, Italy). The human ovarian cancer cell line SKOV‐3 (SK‐OV‐3, RRID: CVCL_0532) was obtained from CLS Cell Lines Service GmbH (Germany), Lot n. 300342‐1420SF. Cells were cultured in DMEM medium supplemented with 10% fetal bovine serum (FBS), 1% penicillin/streptomycin, and 1% glutamine at 37°C in a 5% CO_2_ humidified atmosphere and subcultured twice weekly. Cells were split 1:3 (1 × 10^4^ cells/cm^2^). The cell line was regularly screened for mycoplasma contamination using the MycoAlert™ Mycoplasma Detection Kit (Lonza) and consistently tested negative.

### 2.2. Cytotoxicity Test via MTT Assay

As previously reported for the A2780 cell line (A2780, RRID: CVCL_0134), the inhibition of cellular metabolic activity induced by the four gold compounds (AF, AuTM, CN1, and CN2) in SKOV‐3 cells was evaluated using the MTT (3‐(4,5‐dimethylthiazol‐2‐yl)‐2,5‐diphenyltetrazolium bromide) assay after 72 h of treatment. Briefly, exponentially growing cells were seeded in 96‐well plates at a density of 2 × 10^4^ cells/mL. After 24 h, cells were treated with the compounds in fresh DMEM at concentrations ranging from 0.01 to 100 μM and incubated for 72 h. On the day of the assay, cells were incubated with 0.5 mg/mL MTT for 1 h at 37°C. Following precipitation, blue formazan was dissolved in DMSO, and the optical density was measured in a microplate reader interfaced with the Microplate Manager/PV Version 4.0 software (BioRad Laboratories, Hercules) at 595 nm. From the absorbance measurements, the half‐maximal inhibitory concentration (IC_50_) value of each compound on SKOV‐3 cells was calculated using GraphPad Prism software Version 6.0 (Graphpad Holdings, LLC). All treatments were performed in two independent experiments, each conducted in triplicate. The results are summarized in Table S1, where they are compared with those obtained for the same compounds in A2780 cells.

### 2.3. Sample Collection and Preparation for NMR Analysis

To ensure reproducibility with previously acquired data on A2780 cells, cellular samples for NMR analysis were prepared following an established protocol [8, 38]. SKOV‐3 cells were seeded in 60 cm^2^ tissue culture plates at 80 × 10^3^ cells/mL (total volume 8 mL) and allowed to grow for 24 h. Cells were then treated with each compound at its respective IC_50_ value at 72 h in order to maintain equal toxicity conditions (Table S1). Treatment was stopped at 24 h to capture early‐to‐mid metabolic changes induced by each compound, prior to the onset of significant cell death. Untreated cells were cultured in parallel for the same time and used as controls. At the end of the incubation time, 1 mL of the DMEM medium was collected for the exometabolome analysis. For the endometabolome profiling, cells were washed three times with PBS and then scraped in PBS supplemented with a protease–phosphatase inhibitor cocktail diluted in DMSO (Sigma‐Aldrich). This buffer is used to quench the cellular enzymatic activities and to stabilize the intracellular metabolome. Cells were lysed by sonication on ice and centrifuged at 200, 000*g* for 30 min at 4°C. All samples were stored at −80°C until analysis. For NMR spectral acquisition of cell lysates, frozen samples were thawed and analyzed one at a time to minimize degradation. For sample preparation, 55 μL of D_2_O was added to 495 μL of each lysate. For GM analysis, all samples were thawed and analyzed together using an automatic refrigerated sample changer. For this type of sample, an aliquot of 300 μL of sodium phosphate buffer (70 mM Na_2_HPO_4_; 20% v/v ^2^H_2_O; 4. mM TMSP, pH 7.4) was added to 300 μL of each medium. Both mixtures were homogenized by vortexing for 30 s and transferred into 5 mm NMR tubes (Bruker BioSpin srl).

For each compound, experiments were performed on three independent biological replicates.

### 2.4. NMR Measurements

The NMR‐based metabolomic analysis was performed according to standard procedures. ^1^H NMR spectra were recorded with a Bruker 600 MHz spectrometer (Bruker BioSpin) optimized for metabolomic analysis, operating at 600.13 MHz proton Larmor frequency and equipped with a 5 mm PATXI ^1^H–^13^C–^15^N probe with ^2^H‐decoupling, a *z*‐axis gradient coil, automatic tuning and matching (ATM), and an automatic refrigerated sample changer (SampleJet, Bruker BioSpin). Temperature was stabilized to within 0.1 K with a BTO 2000 thermocouple. Before measurement, to equilibrate the temperature at 300 K, samples were kept for at least 5 min inside the NMR probe head. The spectra of the cell lysate samples were acquired with the Carr–Purcell–Meiboom–Gill (CPMG) sequence using a 1D spin–echo sequence with water presaturation; 512 scans, 73,728 data points, a spectral width of 12,019 Hz, and a relaxation delay of 4 s were used. The spectra of GM were acquired with the CPMG pulse sequence using 64 scans, 73,728 data points, a spectral width of 12,019 Hz, and a relaxation delay of 4 s.

All spectra were acquired with the same receiver gain, indicating similarly efficient water suppression.

The raw data were multiplied by a 0.3 Hz exponential line broadening before applying the Fourier transform. Transformed spectra were automatically corrected for phase and baseline distortions. The spectra were calibrated at the doublet of Ala at 1.49 ppm by using TopSpin 3.6 (Bruker Biospin srl). Spectral quality was assessed by monitoring the linewidth of the alanine CH_3_ resonance at 1.49 ppm, using a linewidth < 1.5 Hz before apodization as the acceptance threshold.

Under these experimental conditions resonances corresponding to a single proton can be reliably detected for metabolites at concentrations of approximately 5 μM or higher.

### 2.5. Data Analysis

A combination of spectral fingerprinting and metabolite profiling was used to analyze the NMR data obtained from cell lysates and GM of SKOV‐3 cells treated with AF, AuTM, CN1, and CN2 together with the corresponding data previously obtained for A2780 cells (six biological replicates for each treatment). All data analyses were performed using the “R” software.

#### 2.5.1. Spectral Fingerprint

Spectral fingerprinting analysis was performed on binned NMR spectra using the R software. To this end, each spectrum of the endo and exometabolome in the 10.00–0.2 ppm region was divided into 0.02 ppm chemical shift bins, and the corresponding spectral areas were integrated using AMIX software. Each bin area was normalized to the total spectral area, calculated excluding the water signal and, for lysates, the signals arising from the quenching cocktail.

Using the binned NMR spectra (hereafter referred to as “Bspectra”), the difference between the areas of the corresponding bins was calculated for each cell line and for both cell lysates and culture media as Bspectrum_treatment − Bspectrum_control for all replicates of each treatment (for a total of 9 replicates, 3 for SKOV‐3 cells and 6 for A2780 cells). Each resulting profile was defined as a Bdifference spectrum, and the average Bdifference spectrum was then obtained as the mean of the corresponding replicate Bdifference_ spectra. In total, 16 average Bdifference_spectra were obtained (8 for the cell lysates—corresponding to AF, AuTM, CN1, and CN2 in both A2780 and SKOV‐3 cells—and another 8 for the corresponding GM). The 8 average Bdifference spectra of cell lysates and the 8 of GM were initially analyzed separately, yielding two distinct data matrices. These were subsequently combined into a single matrix: for each treatment, the average difference spectrum of cell lysates was concatenated with that of the corresponding growth medium to generate an overall metabolic fingerprint of the treatment. The process is summarized in Figure 1A.

**FIGURE 1:**
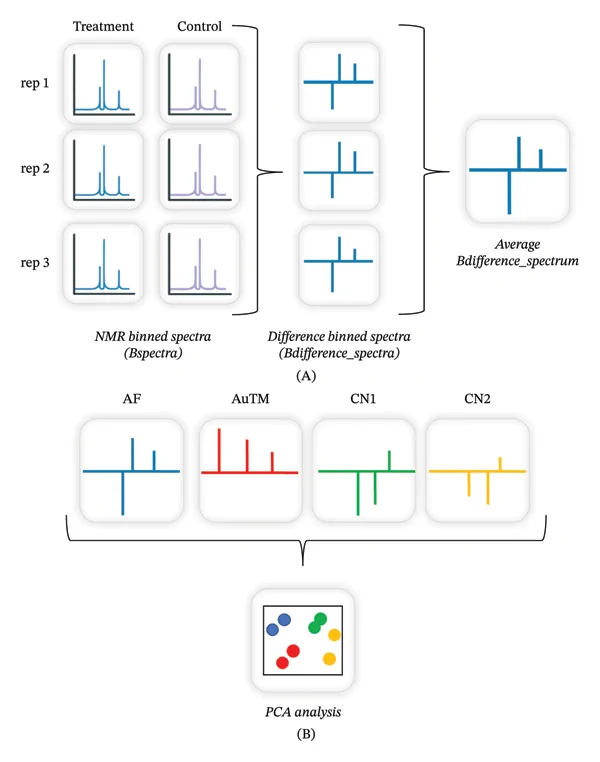
Workflow for NMR fingerprinting and comparative analysis. (A) Schematic representation of the data processing steps used to generate average Bdifference_spectra from binned ^1^H NMR data. (B) PCA‐based clustering obtained from the average Bdifference_spectra, where each point represents a treatment‐specific profile, enabling comparison across treatments.

To obtain an index of cellular dysregulation, the absolute values of all bins in each combined average Bdifference_spectrum (cell lysate plus growth medium) were summed; higher values indicate greater metabolic dysregulation induced by the treatment. Since the calculation was performed on difference spectra, unperturbed metabolic features mathematically canceled out, ensuring that the cumulative sum was heavily driven by genuine biochemical changes rather than baseline fluctuations.

Spectral fingerprints were analyzed using multivariate statistics (Figure 1B). Unsupervised principal component analysis (PCA) was performed using the “prcomp” R function from the “stats” library.

To quantitatively evaluate group separation in the PCA space, a silhouette analysis was conducted based on Euclidean distances using the “silhouette” function from the “cluster” library. Mean silhouette values range from −1 to +1, where values closer to +1 indicate strong cluster cohesion and distinct group separation, while values near or below 0 reflect poor cluster separation. Group centroids were calculated as the mean coordinates of samples within each group. The geometric separation between groups was evaluated by calculating the Euclidean distance between their respective centroids. To statistically assess whether the observed group separation was significant, a Permutational Multivariate Analysis of Variance (PERMANOVA, 100 iterations) was applied using the “adonis2” function from the “vegan” library.

Supervised partial least squares discriminant analysis (PLS‐DA) was performed using the “opls” R function from the “ROpls” library; in all the PLS‐DA models performed, two predictive components were manually specified, as the auto‐fit procedure failed to identify the optimal number of components. The statistical significance and risk of over‐fitting for PLS‐DA models were assessed via permutation testing (200 permutations). PCA and PLS‐DA were applied to both Bspectra and average Bdifference_spectra for comparison purposes.

#### 2.5.2. Metabolite Profiling and Analysis

Metabolites with well‐resolved signals in the spectra were assigned and quantified.

Assignment was performed by using the Chenomx software; the list of assigned metabolites in the ^1^H NMR spectra of A2780 and SKOV‐3 cell lysates (*L*) and GM is reported in Table S2.

The relative quantification of NMR signals was carried out using an in‐house developed R script: for each metabolite a specific integral range (Table S3) was defined based on well‐resolved and isolated resonances, avoiding spectral regions containing overlapping signals. The integration ranges were manually defined based on the spectral assignment and kept constant across all samples. Two different panels of integration ranges were established for cell lysates and GM analysis. The R script calculated the integrated peak area within each predefined spectral region for every spectrum. To account for minor variations in chemical shifts, a spectral alignment step was performed prior to integration, ensuring that the predefined intervals accurately captured the target peaks. The resulting peak areas were normalized to the total spectral area of each spectrum, excluding the residual water resonance and, for cell lysates, the resonances originating from the protease inhibitor cocktail, in order to account for differences in cell number. The normalized peak areas were used as relative metabolite abundances for all downstream statistical analyses.

In the growth medium, metabolites were classified as either taken up or released. This classification was based on comparison of their concentrations at each time point during treatment with their initial levels in the fresh medium. Metabolites showing decreased concentrations were classified as taken up, whereas those showing increased concentrations were classified as released. For released metabolites, lower or higher concentrations during treatment relative to control indicate decreased or increased secretion, respectively. For taken‐up metabolites, lower concentrations relative to controls indicate greater nutrient consumption (i.e., increased uptake), whereas higher concentrations indicate reduced uptake.

The log_2_ fold change (log_2_(FC)) was calculated for each metabolite to describe variations in metabolite levels across treatments. The log_2_(FC) was defined as the median of the log_2_ values of the ratio of the metabolite concentrations of paired samples (treated vs. control). For each treatment, statistically significant metabolites were identified using the nonparametric Wilcoxon–Mann–Whitney test (*p* value < 0.05). The effect size, using the Cliff’s delta formulation, was also calculated to aid in the identification of the meaningful signals, giving an estimation of the magnitude of the separation between the different groups. The magnitude is assessed using the thresholds provided in Romano and Coll [39], that is, |*δ*|<0.147 “negligible (1),” |*δ*|<0.33 “small (2),” |*δ*|<0.474 “medium (3),” otherwise “large (4).” The calculated log_2_(FC), *p* values and Cliff’s delta magnitude ranking are reported in Tables S4–S7. The list of significantly altered metabolites was then used for pathway analysis with MetaboAnalyst 6.0 (https://www.metaboanalyst.ca/). For this analysis, significant metabolites from both the cell lysates and the growth medium were combined. Pathway enrichment was assessed using the hypergeometric test. The importance of metabolites (nodes) within each pathway was estimated based on out‐degree centrality. The reference pathway library was the *Homo sapiens* library from KEGG (Kyoto Encyclopedia of Genes and Genomes, https://www.genome.jp/kegg/pathway.html). Raw pathway enrichment outputs generated by MetaboAnalyst are reported in Tables S8–S15. Pathways that are not functional or biologically relevant in cellular systems were not considered for further analysis together with pathways with *p* value > 0.05 and impact = 0. The impact value is derived from the pathway topology analysis and equals the sum of the importance scores of the metabolites detected in each pathway. As previously described in [32], for each selected enriched pathway an overall pathway FC was calculated, based on the weighted average of the absolute values of the log_2_(FC) for its detected metabolites, using the metabolite importances as weights; the log_2_(FC) absolute values of the metabolites significantly affected in both cell lysates and GM were averaged prior to computing the weighted average for the whole pathway. To highlight the most dysregulated pathways for each treatment in each of the two cell lines, the pathway with the highest FC value was set to 100, and the others were scaled proportionally. Pathway analysis results were visualized using scatter plots.

For each drug treatment, the five most dysregulated pathways that were common to both A2780 and SKOV‐3 cells, together with the associated metabolites that were significantly altered upon treatment, were visualized using a Sankey diagram. The diagram was generated with the “ggalluvial” and “ggplot2” R packages. The input data matrix was structured as a binary presence/absence matrix mapping the annotations of significantly altered metabolites across the selected metabolic pathways.

## 3. Results and Discussion

### 3.1. Spectral Characterization of SKOV‐3 Lysates and Media

The ^1^H NMR spectra of SKOV‐3 lysates and their respective culture media exhibited well‐resolved resonances attributable to central metabolic intermediates, amino acids, glycolytic products, and others. Compared with the previously characterized A2780 cell line, differences in metabolite composition and signal intensities were observed in both the intracellular (endometabolome) and extracellular (exometabolome) compartments (Figures S1 and S2). Accordingly, a clear clustering is observed in the corresponding PCA analyses (Figure S3). A direct comparison of untreated A2780 and SKOV‐3 samples is provided in Table S2, which summarizes the metabolites identified in the spectra of cell lysates and GM across the two cell lines. Accordingly, the number of pathway hits associated with NMR‐detectable metabolites differs between the two cell lines (Table S16). The composition of the culture media (RPMI 1640 and DMEM) also differs; however, irrespective of these differences, the two cell lines show a distinct uptake of the main medium components (Table S17). A2780 cells showed higher consumption of branched‐chain amino acids (Val, Leu, Ile) compared to SKOV‐3 cells, in which uptake was more limited. A similar trend was observed for aromatic amino acids (Phe, Tyr, His), with moderate depletion in A2780 and minimal changes in SKOV‐3, particularly for Phe. Glucose consumption was substantial in both cell lines but more pronounced in A2780 cells. Overall, these results suggest higher metabolic activity in A2780 compared to SKOV‐3 cells. In practice, the above‐discussed baseline differences are expected to significantly influence the variance structure in subsequent multivariate analyses.

### 3.2. Baseline Metabolic Variability Dominates Multivariate Analysis

PCA performed on the full set of raw binned ^1^H NMR spectra (Bspectra) from cell lysates and GM revealed clear separation by cell line, with the different treatment samples overlapping significantly within each cluster (Figure 2A). This was confirmed by strong cluster cohesion by cell line with *s* values larger than 0.55 and *p*‐values from the permutation tests on centroid distances less than 0.05 (Figure 3A,B, right panels; Tables S18 and S19). Conversely, treatment‐related clustering built on AF, AuTM, and CN1/CN2 groups showed poor cohesion (*s* < 0) and nonsignificant centroid separation (*p* values > 0.05), as shown in Figure 3A,B, left panels; Tables S20 and S21. These findings suggest that intrinsic metabolic differences between A2780 and SKOV‐3 cells represent the dominant source of variance in the dataset, effectively masking treatment‐associated metabolic signatures in conventional multivariate analyses. Under these conditions, direct cross‐model comparison of drug‐induced effects is analytically challenging, highlighting a limitation of standard untargeted workflows when applied to heterogeneous biological models, where variance captured by PCA is primarily driven by intrinsic metabolic differences rather than by the experimental perturbation. As a diagnostic control, PLS‐DA models were built to assess whether these methods could bypass the dominant cell‐line variance. However, the models showed no discrimination ability for drug treatments in either cell lysates or GM. Specifically, cross‐validated *Q* ^2^ values were 0.038 (*R* ^2^ *Y* = 0.14) for cell lysates and 0.036 (*R* ^2^ *Y* = 0.15) for media. All the PLS‐DA validation metrics are reported in Table S22. These results confirm that treatment‐related variance is small relative to intrinsic differences between cell lines, preventing robust class discrimination. Collectively, these findings demonstrate that baseline metabolic variability dominates the data structure and obscures treatment effects, thereby motivating the development of a strategy aimed at attenuating baseline contributions prior to multivariate analysis.

**FIGURE 2:**
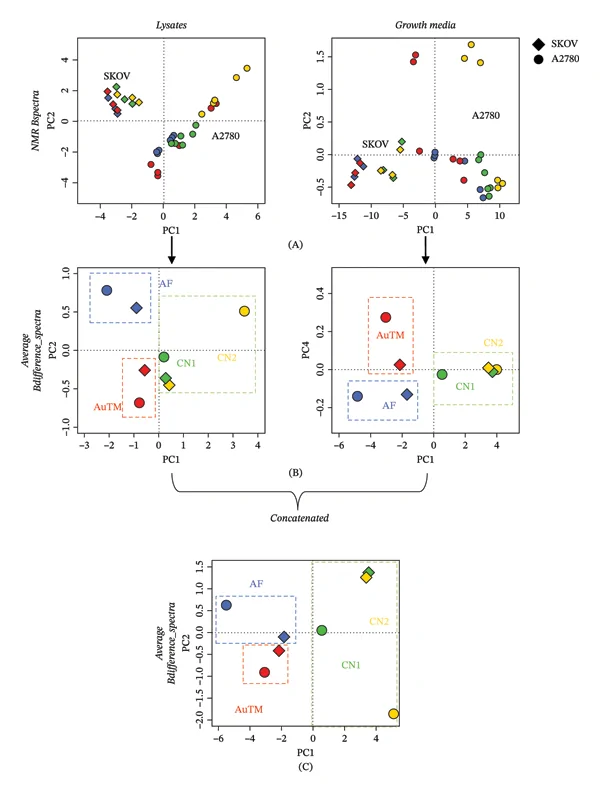
Principal component analysis (PCA) of the NMR profiles. (A) PCA score plots derived from Bspectra of treated cell lysates (left, proportion of variance: PC1 (0.528), PC2 (0.276)) and respective growth media (right, proportion of variance: PC1 (0.985), PC2 (0.0068)). (B) PCA score plots of average Bdifference_spectra of cell lysates (left, proportion of variance: PC1 (0.786), PC2 (0.088)) and growth media (right, proportion of variance: PC1 (0.991), PC2 (0.0048)). (C) PCA score plot of concatenated average Bdifference_spectra (cell lysates + growth media); proportion of variance: PC1 (0.866), PC2 (0.0923). Symbols indicate cell lines: A2780 (circles) and SKOV‐3 (diamonds). Colors indicate treatment: AF (blue); AuTM (red); CN1 (green); and CN2 (yellow).

**FIGURE 3:**
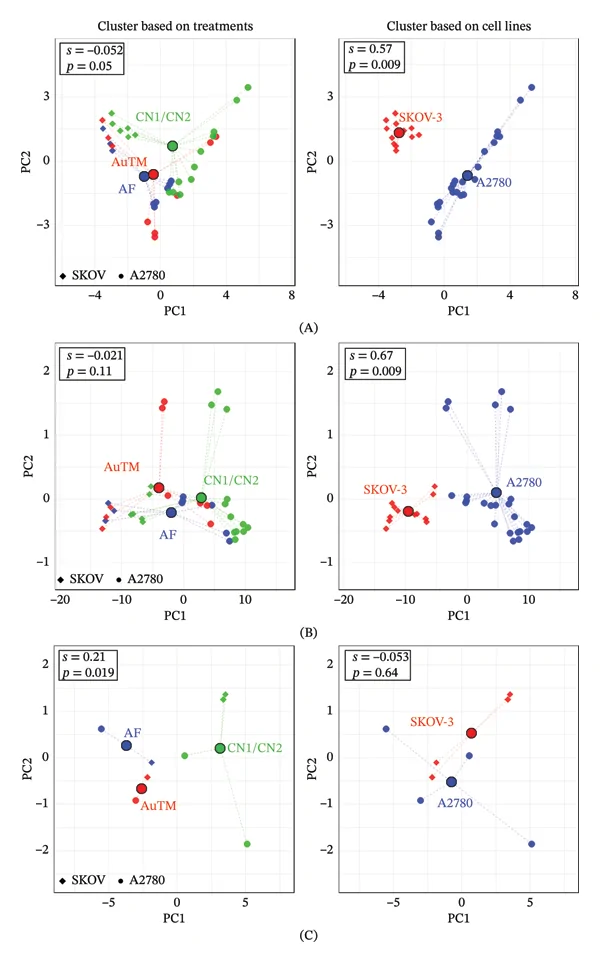
Cluster cohesion analysis of PCA derived from NMR profiles. PCA score plots derived from (A) Bspectra of treated cell lysates; (B) Bspectra of treated growth media; (C) concatenated average Bdifference_spectra (cell lysates + growth media). In each score plot, the cluster centroids are shown as black‐contoured large dots; the respective average silhouette width (*s*) and *p* values from the permutation tests on centroid distances (*p*) are also reported.

### 3.3. Difference‐Spectra Strategy for Comparative Metabolomic Analysis

To mitigate the dominant contribution of baseline variability and enhance the detection of treatment‐related metabolic changes, a difference‐spectra strategy was implemented. Difference Bspectra were obtained by subtracting control Bspectra from treated Bspectra on a matched, replicate‐by‐replicate basis for both cell lysates and GM across the two cell lines. The resulting Bdifference_spectra were then averaged across replicates for each treatment (AF, AuTM, CN1, and CN2), yielding representative profiles for subsequent analysis (Figure 1A).

### 3.4. Multivariate Analysis of Difference Spectra Reveals Drug‐Induced Metabolic Signatures

The resulting average Bdifference_spectra were used as the input for multivariate analysis. PCA performed on both cell lysates and GM showed a markedly different clustering pattern compared to the raw spectral dataset (Figure 2B). In contrast to the PCA of the original Bspectra (Figure 2A), analysis of the Bdifference_spectra enabled clearer visualization of treatment‐related metabolic signatures and allowed direct comparison of drug‐induced effects across the two cell lines. Combining lysate and medium data further improved the discrimination among the different compounds (Figure 2C). Baseline cell‐line differences were successfully subtracted; AF, AuTM, and the two carbene compounds (CN1 and CN2) clearly show three different clusters. In fact, very low cluster cohesion with *s* < 0 and *p* > 0.05 was obtained for cell‐line clustering, while for treatment‐related clusters, the cohesion significantly improved up to *s* = 0.22 and a statistically significant separation was achieved (*p* < 0.05), Figure 3C, Tables S23, and S24.

### 3.5. Quantification and Comparison of Metabolic Perturbations

To compare the overall metabolic impact of the different compounds, dysregulation indexes were calculated from the Bdifference_spectra, as detailed in Section 2.5.1. These indexes provide a quantitative measure of the extent of metabolic perturbation induced by each treatment and capture the cumulative magnitude of both up and downregulated metabolic variations, preventing opposite metabolic shifts from neutralizing each other. The calculated dysregulation index values are on average higher for A2780 cells than for SKOV‐3 cells, despite the fact that the required dosage of each drug to achieve equal toxicity is always lower for A2780 cells (Table S1). These indicate a greater metabolic sensitivity of A2780 to the treatments. Despite these differences in the magnitude of the responses, the relative ranking of the compounds was largely conserved between A2780 and SKOV‐3 cells (Figure 4A).

**FIGURE 4:**
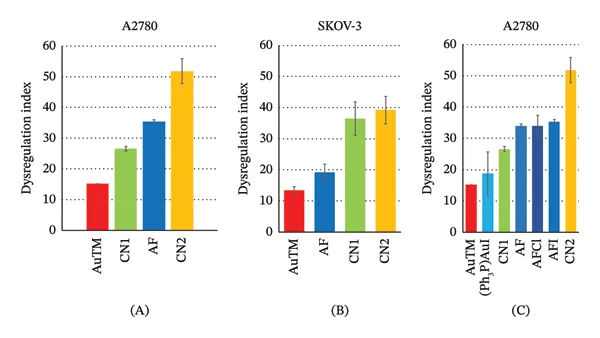
Comparative analysis of metabolic dysregulation induced by the four selected gold(I) compounds and AF‐analogs. (A) Bar plots representing the dysregulation indices (average ± sd) for AF, AuTM, CN1, and CN2 in A2780 (left) and SKOV‐3 (right) cells. (C) Bar plots of the dysregulation indices (average ± sd) for all compounds in A2780 cells. Colors indicate treatments: AF (blue); AuTM (red); CN1 (green); CN2 (yellow); and AF‐analogs (AFCl, AFI, (Ph_3_P)AuI) shown as shades of blue.

### 3.6. Validation of the Difference‐Spectra Approach Using A2780 Cells Treated With AF Analogs

Previously acquired metabolomic data from A2780 cells treated with three AF analogs were used to further assess the robustness of the proposed approach. These included the chloro(triethylphosphine)gold(I) (AFCl), the iodide(triethylphosphine)gold(I) (AFI), in both of which the thiosugar ligand is replaced by chloride or iodide while retaining the triethylphosphine moiety, and the iodide(triphenylphosphine)gold(I) (Ph_3_P)AuI, in which the triethylphosphine ligand is replaced by the bulkier triphenylphosphine (Figure S4). The spectra were processed according to the difference‐spectra procedure described above (Figure 1), and the resulting Bdifference_spectra were subjected to PCA. The analysis (Figure 5) confirmed the ability of the approach to enhance the detection of treatment‐related differences among closely related compounds. Projection of these compounds in the PCA space defined by the Bdifference_spectra showed that they clustered in close proximity to AF (Figure 5A–B) with Euclidean distances of less than 1 with respect to the AF centroid, indicating similar metabolic fingerprints, consistent with observations obtained using the conventional approach [32]. When they were considered as belonging to the AF cluster, the average *s* value increased up to 0.3 with a *p* value = 0.009 (Figure 5C). This result demonstrates that the difference‐spectra strategy can reliably position compounds within a shared metabolic response landscape. Moreover, the dysregulation analysis reported in Figure 4B confirmed a metabolic perturbation comparable to that induced by AF in A2780 cells.

FIGURE 5Validation of the difference‐spectra approach using A2780 cells treated with AF analogs. PCA derived from concatenated average Bdifference_spectra (cell lysates + growth media). (A) PCA score plot (proportion of variance: PC1 (0.871), PC2 (0.0556)); colors indicate treatments: AF (blue); AuTM (red); CN1 (green); CN2 (yellow); and AF‐analogs (AFCl, AFI, (Ph_3_P)AuI) shown as shades of blue. (B) PCA score plot with cluster centroids shown as black‐contoured large dots and the respective 6‐group Euclidean distance matrix. (C) PCA score plot with cluster centroids shown as black‐contoured big dots and the respective 3‐group Euclidean distance matrix (the three AF analogs are considered as belonging to the AF group); the average silhouette width (*s*) and *p* values from the permutation tests on centroid distances (*p*) are also reported.

**Figure:**
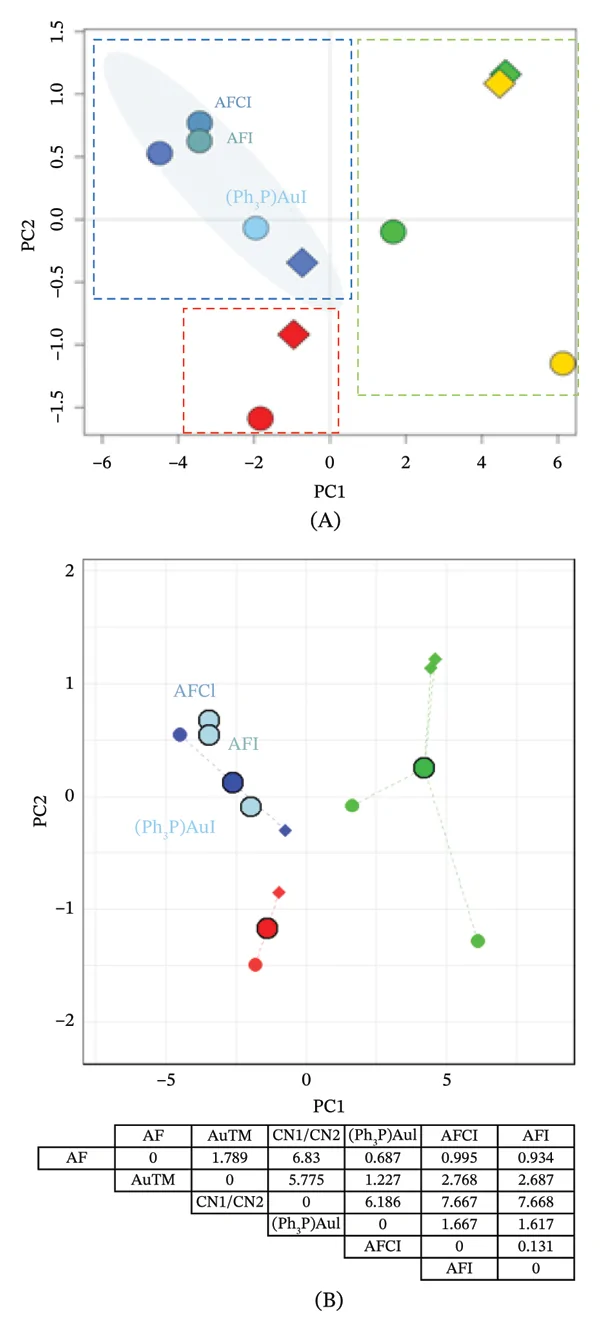

**Figure:**
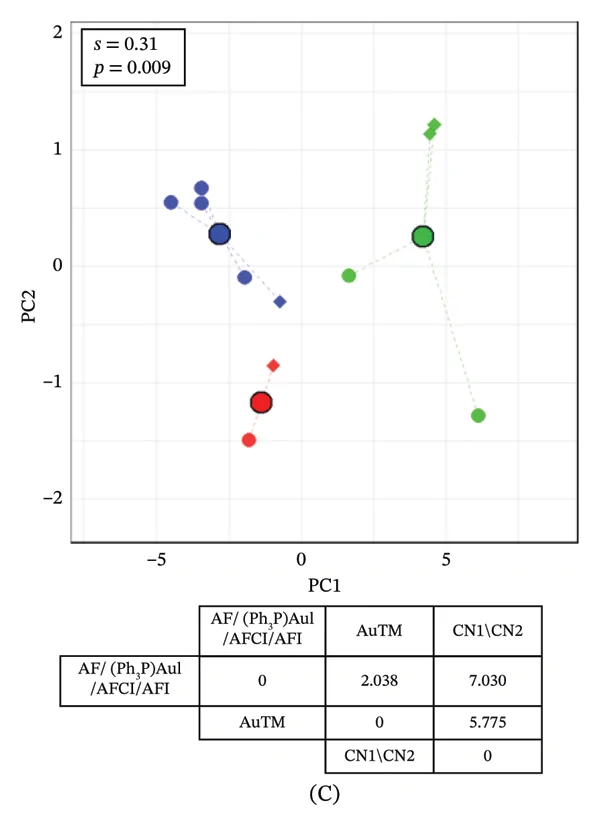

### 3.7. Validation of the Difference‐Spectra Approach Using Comparative Pathway Analysis of Drug‐Induced Metabolic Alterations

To validate the observed treatment‐related clustering using the concatenated average Bdifference_spectra, independently of the cell line, pathway analysis was used to highlight treatment‐specific metabolic responses in A2780 and SKOV‐3 cells (Figure 6). In order not only to obtain a list of affected pathways but also to highlight those most dysregulated by each treatment in each cell line, we considered both the number of statistically significant metabolites and their measured fold changes (Tables S4–S7), as previously reported in [32]. Enriched pathways are shown as dot plots, where color intensity and dot size reflect the weighted average of the absolute log_2_(FC) of the detected metabolites within each pathway. To enable comparison across treatments and cell lines, pathway responses were normalized by setting the highest fold change within each condition to 100 and scaling all other pathways accordingly. This representation enabled a direct comparison of the relative impact of each treatment on metabolic pathways, revealing a largely conserved set of perturbed pathways across the two cell lines, with treatment‐dependent differences in their modulation. The most affected pathways included glutathione metabolism for AF, the TCA cycle for AuTM, and glycolysis and galactose metabolism for both CN1 and CN2, highlighting the consistent involvement of key metabolic processes across treatments.

**FIGURE 6:**
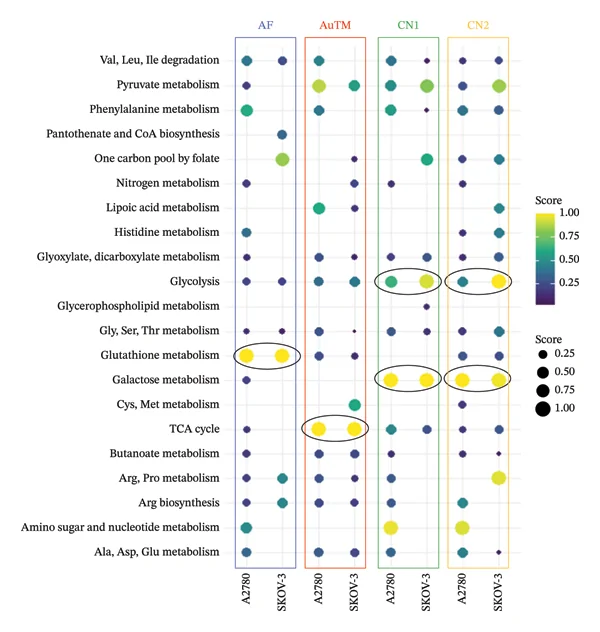
Dot plots of the pathway analysis results for the different treatments in A2780 and SKOV‐3 cells. Each enriched pathway is represented as a dot with color intensity and size reflecting the weighted average of the absolute values of the log2(FC) for the detected metabolites within each pathway, using metabolite importance as a weight. For each treatment and cell line, the pathway with the highest FC value was set to 100, and all other pathways were scaled proportionally.

### 3.8. Mechanistic Interpretation

To illustrate the biological interpretation of the results, a detailed analysis was performed for AF. Integration of pathway enrichment data with the corresponding metabolite changes highlighted the central role of glutathione metabolism (Figure 7A). Consistent with this finding, intracellular levels of glutathione and related metabolites showed significant changes in both A2780 and SKOV‐3 cells upon treatment, supporting a mechanism associated with an oxidative stress response (Figure 7B). The other most dysregulated pathways emerging for AF (Figure 7A) and shared between A2780 and SKOV‐3 cells include arginine biosynthesis, glycolysis, and the metabolism of several amino acids: Arg/Pro and Val/Leu/Ile. These pathways are supported by a largely overlapping, but not identical, set of metabolites, as shown in Figure 7A and detailed in Table S2.

**FIGURE 7:**
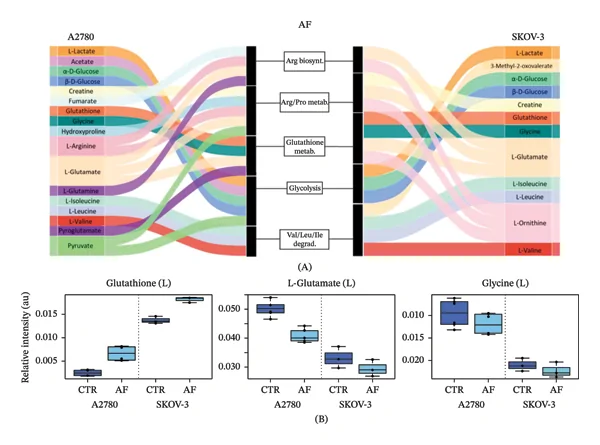
Integrated pathway and metabolite analysis of AF‐induced metabolic changes. (A) Sankey diagram showing the five most dysregulated pathways shared between A2780 and SKOV‐3 cells upon AF treatment, together with the associated metabolites. (B) Box plots of relative metabolite concentrations in the intracellular compartment (cell lysates, L). Intracellular levels of glutathione, glutamate, and glycine are shown for control (blue) and AF‐treated (cyan) A2780 and SKOV‐3 cells.

Similar pathway–metabolite relationships were observed for the other compounds and are detailed in the Supporting Information (Figures S5–S7). For AuTM in particular, the affected pathways include not only the citrate cycle but also alanine, aspartate, and glutamate metabolism, glycolysis, pyruvate metabolism, and lipoic acid metabolism (Figures 6 and S5). Notably, pyruvate contributes to all five pathways in both cell lines. For CN1 and CN2, in addition to the marked similarity in glycolysis and galactose metabolism, as well as the common involvement of pyruvate metabolism (Figures 6, S6, and S7), some notable differences emerge. The TCA cycle and Gly/Ser/Thr metabolism are specifically associated with CN1, whereas phenylalanine metabolism and glutathione metabolism are characteristic of CN2.

### 3.9. Potential Limitations and Scope of the Workflow

While the proposed difference‐spectra strategy successfully attenuates dominant baseline metabolic variance to facilitate drug screening, some limitations should be considered. By design, the subtraction procedure focuses on capturing the shared, systemic metabolic response of the compounds across different biological backgrounds. Consequently, it may partially obscure specific treatment‐by‐cell line interaction effects or responses that are strictly dependent on a unique cellular environment. However, it is important to note that this workflow is intended as a rapid, high‐throughput multivariate screening tool to classify modes of action, rather than an exhaustive mapping of cell‐specific biology. Once the global screening and clustering are achieved, downstream univariate testing and metabolite‐level analyses remain essential to dissect the specific biological fingerprints and extract the unique metabolic adaptations of each cell line.

## 4. Conclusion

Comparative metabolomics across distinct cell lines poses significant analytical challenges. Baseline metabolic differences can dominate multivariate variance and obscure treatment‐associated signatures, a limitation not routinely addressed in standard workflows. By redefining the variance structure prior to multivariate modeling, the proposed difference‐spectra strategy provides a practical means to disentangle baseline heterogeneity from treatment‐driven effects, thereby extending the applicability of untargeted NMR metabolomics to drug screening in heterogeneous biological systems. Crucially, this approach enables effective, high‐throughput drug screening based purely on global multivariate fingerprints, allowing the identification of shared modes of action without the need for time‐consuming single‐metabolite quantification or pathway enrichment analysis.

To validate the biological accuracy of the clustering achieved through this multivariate workflow, downstream univariate testing and comparative pathway analyses were performed. These validation steps confirmed the identification of compound‐specific metabolic signatures across the two cell lines. Distinct patterns of pathway perturbation were observed for the different compounds, indicating divergent metabolic responses. Specifically, AF predominantly affected glutathione metabolism, AuTM perturbed central carbon metabolism, and CN1 and CN2 were mainly associated with changes in glycolysis and extracellular metabolic pathways, with additional compound‐specific differences. The use of multiple cell lines increases confidence in the biological relevance of the observed metabolic alterations that extends beyond the most dysregulated pathways.

Overall, this study highlights the effectiveness of difference‐spectra methodology as an independent screening tool that can accurately classify drug mechanisms directly from untargeted multivariate profiles.

## Funding

This work was supported by the Associazione Italiana per la Ricerca sul Cancro, IG 2021 ID 26169; Ministero dell’Università e della Ricerca, DICUS2.0; Ministero della Salute, PAN‐HUB 2021‐T4‐AN‐07. Open access publishing facilitated by Università degli Studi di Firenze, as part of the Wiley–CRUI‐CARE agreement.

## Conflicts of Interest

The authors declare no conflicts of interest.

## Supporting Information

Additional supporting information can be found online in the Supporting Information section.

## Supporting information


**Supporting Information** Supporting 1. Supporting Figures: Figure S1: Representative ^1^H NMR spectra of SKOV‐3 and A2780 cell lysates; Figure S2: Representative ^1^H NMR spectra of SKOV‐3 and A2780 GM. Figure S3: PCA score plots of untreated A2780 and SKOV‐3 cells. Figure S4: AF, AFCl, AFI, and (Ph_3_P)AuI chemical structures. Figure S5. Integrated pathway and metabolite analysis of AuTM‐induced metabolic changes. Figure S6. Integrated pathway and metabolite analysis of CN1‐induced metabolic changes. Figure S7. Integrated pathway and metabolite analysis of CN2‐induced metabolic changes. Supporting 2. Supporting Tables: Table S1: IC_50_ values (μM). Table S2: List of metabolites identified in the ^1^H NMR spectra of A2780 and SKOV‐3 cells. Table S3. List of metabolites quantified with the respective integration ranges. Table S4. List of metabolites analyzed in the cell lysates of SKOV‐3 cells with log_2_(FC), *p* values and Cliff’s Delta magnitude ranking. Table S5. List of metabolites analyzed in the GM of SKOV‐3 cells with log_2_(FC), *p* values and Cliff’s Delta magnitude ranking. Table S6. List of metabolites analyzed in the cell lysates of A2780 cells with log_2_(FC), *p* values and Cliff’s Delta magnitude ranking. Table S7. List of metabolites analyzed in the GM of A2780 cells with log_2_(FC), *p* values and Cliff’s Delta magnitude ranking. Table S8. Pathway enrichment output for AF treatment in SKOV‐3 cells. Table S9. Pathway enrichment output for AuTM treatment in SKOV‐3 cells. Table S10. Pathway enrichment output for CN1 treatment in SKOV‐3 cells. Table S11. Pathway enrichment output generated for CN2 treatment in SKOV‐3 cells. Table S12. Pathway enrichment output for AF treatment in A2780 cells. Table S13. Pathway enrichment output for AuTM treatment in A2780 cells. Table S14. Pathway enrichment output for CN1 treatment in A2780 cells. Table S15. Pathway enrichment output for CN2 treatment in A2780 cells. Table S16: List of metabolic pathways (KEGG). Table S17: Concentrations (mg/L) of representative nutrients in A2780 and SKOV‐3 cell culture media. Table S18. Cluster analysis based on cell lines of PCA score plot derived from Bspectra of treated cell lysates. Table S19. Cluster analysis based on cell lines of PCA score plot derived from Bspectra of treated GM. Table S20. Cluster analysis based on treatments of PCA score plot derived from Bspectra of treated cell lysates. Table S21. Cluster analysis based on treatments of PCA score plot derived from Bspectra of treated cell lysates. Table S22. Statistical validation and quality parameters for supervised (PLS‐DA) multivariate models. Table S23. Cluster analysis based on cell lines of PCA score plot derived from concatenated average Bdifference_spectra (cell lysates +  GM). Table S24. Cluster analysis based on treatments of PCA score plots derived from concatenated average Bdifference_spectra (cell lysates +  GM).

## Data Availability

The data that support the findings of this study are available from the corresponding author upon reasonable request.
