# Supplementary material for: Identifying Drug‐Induced Metabolic Signatures in Comparative Untargeted NMR Metabolomics Using Difference Spectra
Source: Bioinorg Chem Appl. 2026 Oct 23;2026:9636071. doi: 10.1155/bca/9636071 (PMC13691625; doi:10.1155/bca/9636071)
Supplement: Supplementary file 1 [file BCA-2026-9636071-s001.docx]

Identifying Drug-Induced Metabolic Signatures in Comparative Untargeted NMR Metabolomics Using Difference Spectra

*Giorgio Di Paco^1^*, Veronica Ghini^1^*, Michele Mannelli^2^, Tania Gamberi^2^, Antonio Rosato^1,3^, Luigi Messori^1^, Paola Turano^1,3^*

^1^Department of Chemistry "Ugo Schiff", University of Florence, Sesto Fiorentino 50019, Italy.

^2^ Department of Experimental and Clinical Biomedical Sciences "Mario Serio", University of Florence, Florence 50134, Italy.

^3^Magnetic Resonance Center (CERM), University of Florence, Sesto Fiorentino 50019, Italy.

*Authors contributed equally

Supporting information

**Figure S1**. Representative ^1^H NMR spectra of SKOV-3 (black trace) and A2780 (red trace) cell lysates. A) Up-field and B) down-field spectral regions. The asterisk (*) indicates signals originating from the protease and phosphatase inhibitor cocktail. Metabolites labelled in black were quantified in both cell lines while those quantified in only one cell line are labelled in cyan above the corresponding spectrum. For the sake of visual clarity, only one or two resonances per metabolite are annotated; the complete list of assigned signals is provided in Table S2.

**Figure S2**. Representative ^1^H NMR spectra of SKOV-3 (black trace) and A2780 (red trace) growth media. For comparison, spectra of the respective blank media, DMEM (blue trace) and RPMI 1640 (green trace), are also shown. A) Up-field and B) down-field spectral regions. Metabolites labelled in black were quantified in both cell lines, while those quantified in only one cell line are labelled in cyan above the corresponding spectrum. For the sake of visual clarity, only one or two resonances per metabolite are annotated; the complete list of assigned signals is provided in Table S2.

**Figure S3**. PCA score plots derived from Bspectra of cell lysates (left, proportion of variance: PC1 (0.650), PC2 (0.212)) and corresponding growth media (right, proportion of variance: PC1 (0.990), PC2 (0.0072)) of untreated A2780 cells (dots) and SKOV-3 cells (diamonds).

**Figure S4**. Auranofin (AF), chloro(triethylphosphine)gold(I) (AFCl), iodide(triethylphosphine)gold(I) (AFI), and iodide(triphenylphosphine)gold(I) (Ph₃P)AuI chemical structures, with leaving groups highlighted. The IC_50_ values determined in A2780 cells are also reported.

**Figure S5**. Integrated pathway and metabolite analysis of AuTM-induced metabolic changes. A) Sankey diagram showing the five most dysregulated pathways shared between A2780 and SKOV-3, upon AuTM treatment, together with the associated metabolites. B) Box plots of relative metabolite concentrations in intracellular (cell lysates, L) and extracellular (growth media, GM) metabolome. Intracellular levels of glutamate, succinate, fumarate and extracellular levels of pyruvate and succinate are shown for control (red) and AuTM-treated (salmon) A2780 and SKOV-3 cells.

**Figure S6**. Integrated pathway and metabolite analysis of CN1-induced metabolic changes. A) Sankey diagram showing the five most dysregulated pathways shared between A2780 and SKOV-3 cells upon CN1 treatment, together with the associated metabolites. B) Box plots of relative metabolite concentrations in the extracellular compartment (growth media, GM). Extracellular levels of pyruvate, lactate and glucose are shown for control (forest green) and CN1-treated (light green) A2780 and SKOV-3 cells.

**Figure S7**. Integrated pathway and metabolite analysis of CN2-induced metabolic changes. A) Sankey diagram showing the five most dysregulated pathways shared between A2780 and SKOV-3 cells upon CN2 treatment, together with the associated metabolites. B) Box plots of relative metabolite concentrations in the extracellular compartment (growth media, GM). Extracellular levels of pyruvate, lactate and glucose are shown for control (dark yellow) and CN2-treated (light yellow) A2780 and SKOV-3 cells.

**Table S1**. IC_50_ values (μM) determined by MTT assay after 72 h exposure.

|  | AF | AuTM | CN1 | CN2 |
| --- | --- | --- | --- | --- |
| A2780 | 0.8 ± 0.2 | 47 ± 2 | 3.5 ± 0.9 | 0.1 ± 0.01 |
| SKOV-3 | 2.6 ± 0.4 | 60 ± 10 | 16 ± 1.9 | 1.0 ± 0.2 |

**Table S2**. List of metabolites identified in the ^1^H NMR spectra of A2780 and SKOV-3 cell lysates (L) and growth media (GM), as assigned using the Chenomx software. Chemical shift (ppm) and signal multiplicity (s= singlet; d= doublet; t= triplet; q= quartet; dd= doublet of doublets; m= multiplet) are reported. The corresponding Human Metabolome Database (HMDB) ID code is also provided for each entry.

| Metabolite | L | GM | L | GM | ^1^H chemical shift (ppm) and signal multiplicity | HMDB ID |
| --- | --- | --- | --- | --- | --- | --- |
|  | A2780 | | SKOV-3 | |  |  |
| 1-methylnicotinamide |  |  | X |  | 9.27(s); 8.96(d); 8.89(d);8.17(m),4.47(s) | HMDB0000699 |
| 3-hydroxyisovalerate (3-HIA) |  | X |  |  | 2.35 (s); 1.26 (s) | HMDB0000754 |
| 3-methyl-2-oxovalerate (KMV) |  | X |  | X | 2.93 (m); 1.70 (m); 1.46 (m); 1.11 (d); 0.88 (t) | HMDB0000491 |
| 4-hydroxyproline | X |  |  |  | 4.67 (m); 4.33 (dd); 3.49 (m); 3.37 (m); 2.43 (m); 2.14 (m) | HMDB0000725 |
| acetate | X | X | X | X | 1.925 (s) | HMDB0000042 |
| acetone | X | X | X |  | 2.22 (s) | HMDB0001659 |
| L-alanine | X | X | X | X | 3.78 (q); 1.485 (d) | HMDB0000161 |
| L-arginine |  | X |  | X | 3.76 (t); 3.24 (t); 1.90 (m); 1.68 (m) | HMDB0000517 |
| L-asparagine | X | X |  |  | 4.00 (dd); 2.95 (m); 2.84 (m) | HMDB0000168 |
| L-aspartate |  | X |  |  | 3.89 (dd); 2.81 (m); 2.66 (m) | HMDB0000191 |
| AXP (AMP/ADP/ATP) | X |  | X |  | 8.60 (s) | - |
| choline | X | X | X | X | 4.06 (m); 3.51 (m); 3.21 (s) | HMDB0000097 |
| citrate | X | X |  | X | 2.68 (d); 2.55 (d) | HMDB0000094 |
| creatine | X |  | X |  | 3.93 (s); 3.04 (s) | HMDB0000064 |
| cystine |  | X |  |  | 4.10 (dd); 3.36 (dd); 3.17 (m) | HMDB0000192 |
| formate |  | X |  | X | 8.445 (s) | HMDB0000142 |
| fumarate | X | X | X | X | 6.52 (s) | HMDB0000134 |
| D-glucose  (α-D-glucose + β-D-glucose) |  | X | X | X | 5.23 (d); 4.64 (d); 3.89 (dd); 3.82 (m); 3.72 (m); 3.53 (dd); 3.48 (t); 3.45 (m) 3.40 (m); 3.24 (dd) | HMDB0000122 |
| glucose-1-phosphate | X |  |  |  | 5.44 (dd); 3.89 (m); 3.78 (t); 3.74 (dd); 3.48 (m); 3.4 (dd) | HMDB0001586 |
| L-glutamate | X | X | X | X | 3.76 (dd); 2.36 (m); 2.11 (m); 2.05 (m) | HMDB0000148 |
| L-glutamine | X | X |  |  | 3.77 (t); 2.46 (m); 2.14 (m) | HMDB0000641 |
| glutathione | X |  | X |  | 8.25 (m); 4.58 (m); 3.77 (m); 2.96 (m) ;2.5 6 (m); 2.16 (m) | HMDB0000125 |
| glycerophosphocholine (GPC) | X |  | X |  | 4.30 (m); 3.90 (m); 3.64 (m); 3.23 (s) | HMDB0000086 |
| glycine | X | X | X | X | 3.57 (s) | HMDB0000123 |
| GXP (GTP/GDP) | X |  |  |  | 5.94 (d) | - |
| L-histidine | X | X | X | X | 7.8 (d); 7.05 (d); 3.99 (dd); 3.23 (m); 3.16 (m) | HMDB0000177 |
| inosine |  |  | X |  | 8.3 (s); 8.19 (s); 6.05 (d); 4.80 (s); 4.42 (dd); 4.26 (dd); 3.90 (dd); 3.83 (dd) | HMDB0000195 |
| inosine monophosphate (IMP) | X |  | X |  | 8.54 (s); 8.21 (s); 6.14 (d); 4.5 (m); 4.36 (m); 4.02 (m) | HMDB0000175 |
| L-isoleucine | X | X | X | X | 3.68 (d); 1.97 (m); 1.46 (m); 1.25 (m); 1.01 (d); 0.94 (t) | HMDB0000172 |
| L-lactate | X | X | X | X | 4.12 (q); 1.33 (d) | HMDB0000190 |
| L-leucine | X | X | X | X | 3.73 (m); 1.70 (m); 0.96 (t) | HMDB0000687 |
| L-lysine |  | X | X | X | 3.74 (t); 3.02 (t); 1.89 (m); 1.71 (m); 1.46 (m) | HMDB0000182 |
| L-methionine |  | X |  | X | 3.85 (dd); 2.63 (t); 2.16 (m) | HMDB0000696 |
| myo-inositol | X | X | X | X | 4.07 (t); 3.63 (t); 3.54 (m); 3.29 (t) | HMDB0000211 |
| niacinamide | X |  | X | X | 8.95 (dd); 8.72 (dd); 8.24 (m); 7.58(m) | HMDB0001406 |
| ornithine |  | X |  | X | 3.76 (t); 3.05 (t); 1.94 (m); 1.82 (m); 1.73 (m) | HMDB0000214 |
| L-phenylalanine | X | X | X | X | 7.44 (m); 7.37 (m); 3.99 (dd); 3.27 (m); 3.11 (m) | HMDB0000159 |
| phosphocholine (PC) | X |  | X |  | 4.17 (m); 3.59 (m); 3.22 (s) | HMDB0001565 |
| phosphocreatine |  |  | X |  | 3.94 (s); 3.04 (s) | HMDB0001511 |
| L-proline |  | X | X |  | 4.12 (dd); 3.41 (m); 3.33 (m); 2.34 (m); 2.06 (m); 2.00 (m) | HMDB0000162 |
| pyroglutamate |  | X |  | X | 4.16 (dd); 2.50 (m); 2.39 (m); 2.02 (m) | HMDB0000267 |
| pyruvate |  | X |  | X | 2.37 (s) | HMDB0000243 |
| L-serine |  | X |  | X | 3.96 (m); 3.83 (dd) | HMDB0000187 |
| succinate | X | X | X | X | 2.41 (s) | HMDB0000254 |
| taurine | X |  |  |  | 3.42 (t); 3.26 (t) | HMDB0000251 |
| L-threonine |  |  | X | X | 4.25 (m); 3.58 (d); 1.32 (d) | HMDB0000167 |
| L-tryptophan |  |  |  | X | 7.72 (d); 7.53 (d); 7.31 (s); 7.27 (m); 7.19 (m); 4.05(dd); 3.47 (dd); 3.30 (dd) | HMDB0000929 |
| L-tyrosine | X | X | X | X | 7.20 (m); 6.91 (m); 3.93 (dd); 3.2 (m); 3.06 (m) | HMDB0000158 |
| UDP-N-acetylglucosamine (UDP-GlcNAc) | X |  |  |  | 8.3 (d); 7.95 (d); 5.99 (d); 5.97 (d); 5.52 (dd); 4.36 (m); 4.28 (m); 4.23 (m); 4.17 (m); 3.98 (m); 3.92 (m); 3.87 (m); 3.5 (dd); 2.1 (s) | HMDB0000290 |
| UDP-N-acetylgalactosamine (UDP-GalNAc) | X |  |  |  | 7.95 (d); 5.97 (d); 5.97 (d); 5.55 (dd); 4.37 (dd); 4.25 (m); 4.2 (m); 4.1 (dd); 4.01 (dd); 3.85 (dd); 3.83 (dd); 3.83 (m); 3.74 (dd); 3.69 (dd); 3.49 (m); 2.66 (s) | HMDB0000304 |
| UDP-sugar | X |  |  |  | 5.61 (dd) | - |
| uracil |  |  | X |  | 7.53 (d); 5.79 (d) | HMDB0000300 |
| uridine |  |  | X |  | 7.86 (d); 5.90 (d); 5.88 (d); 4.34 (dd); 4.22 (t); 4.12 (ddd); 3.91 (dd); 3.80 (dd) | HMDB0000296 |
| L-valine | X | X | X | X | 3.60 (d); 2.26 (m); 1.045 (d); 0.99 (d) | HMDB0000883 |

**Table S3**. List of metabolites quantified in the ^1^H NMR spectra of A2780 and SKOV-3 cell lysates (L) and growth media (GM), with the respective integration ranges used for relative quantification. nq= assigned but not quantified. For metabolites identified in both A2780 and SKOV-3 cells, the reported integration ranges refer to the A2780 spectra; minor adjustments to the integration windows may have been applied to the SKOV-3 spectra to account for slight baseline or peak alignment variations.

| Metabolite | L | GM | L | GM | integration range (L) | Integration range (GM) |
| --- | --- | --- | --- | --- | --- | --- |
|  | A2780 | | SKOV-3 | |  |  |
| 1-methylnicotinamide |  |  | X |  | 9.2903-9.2744 | - |
| 3-hydroxyisovalerate |  | X |  |  | - | 1.2554-1.2504 |
| 3-methyl-2-oxovalerate |  | X |  | X | - | 1.1018-1.0892 |
| 4-hydroxyproline | X |  |  |  | 3.4900-3.4720 | - |
| acetate | X | X | X | X | 1.9260-1.9190 | 1.9239-1.9170 |
| acetone | nq | X | X | nq |  | 2.2390-2.2336 |
| L-alanine | X | X | X | X | 1.5050-1.4700 | 1.4950-1.4842 |
| L-arginine |  | X |  | nq | - | 1.6940-1.6325 |
| L-asparagine | X | X |  |  | 2.8840-2.8780 | 2.9467-2.9275 |
| L-aspartate |  | X |  |  | - | 2.8096-2.8027 |
| AXP (AMP/ADP/ATP) | X |  | X |  | 8.6120-8.5900 | - |
| choline | X | X | X | X | 3.2083-3.2036 | 3.2044-3.1988 |
| citrate | X | X |  | X | 2.5766-2.5725 | 2.5710-2.5624 |
| creatine | X |  | X |  | 3.9420-3.9270 | - |
| cystine |  | X |  |  | - | 3.1945-3.1600 |
| formate |  | X |  | X | - | 8.4650-8.4590 |
| fumarate | X | X | X | X | 6.5280-6.5170 | 6.5263-6.5215 |
| D-glucose  (α-D-glucose + β-D-glucose) |  | X | X | X | 5.2496-5.2344 | 5.2508-5.2353 |
| glucose-1-phosphate | X |  |  |  | 5.4770-5.4491 | - |
| L-glutamate | X | X | X | X | 2.3800-2.3340 | 2.3537-2.3419 |
| L-glutamine | X | X |  |  | 2.4820-2.4740 | 2.4723-2.4390 |
| glutathione | X |  | X |  | 2.5620-2.5390 | - |
| glycerophosphocholine (GPC) | X |  | X |  | 3.2360-3.2290 | - |
| glycine | X | X | X | X | 3.5720-3.5630 | 3.5722-3.5631 |
| GXP (GTP/GDP) | X |  |  |  | 5.9520-5.9230 | - |
| L-histidine | X | X | X | X | 7.1050-7.0830 | 7.8011-7.7870 |
| inosine |  |  | X |  | 8.2412-8.2350 | - |
| inosine monophosphate (IMP) | X |  | nq |  | 6.1590-6.1320 | - |
| L-isoleucine | X | X | X | X | 1.0250-1.0150 | 1.0240-1.0120 |
| L-lactate | X | X | nq | X | 4.1190-4.1080 | 1.3430-1.3360 |
| L-leucine | X | X | X | X | 0.9820-0.9710 | 0.9800-0.9589 |
| L-lysine |  | X | X | X | 1.9062-1.8966 | 3.0290-3.0220 |
| L-methionine |  | X |  | X | - | 2.6427-2.6329 |
| myo-inositol | X | X | X | X | 4.080-4.0610 | 3.6405-3.6310 |
| niacinamide | X |  | X | X | 8.7280-8.7100 | 8.9540-8.9418 |
| ornithine |  | X |  | X | - | 3.0845-3.0742 |
| L-phenylalanine | X | X | X | X | 7.4038-7.3930 | 7.3404-7.3301 |
| phosphocholine (PC) | X |  | X |  | 3.2280-3.2170 | - |
| L-proline |  | X | X |  | 2.0106-1.9997 | 3.3390-3.3330 |
| pyroglutamate |  | X |  | X | - | 4.1874-4.1722 |
| pyruvate |  | X |  | X | - | 2.3790-2.3732 |
| L-serine |  | X |  | X | - | 3.9684-3.9605 |
| succinate | X | X | X | X | 2.4130-2.4040 | 2.4078-2.4053 |
| taurine | X |  |  |  | 3.2790-3.2734 | - |
| L-threonine |  |  | X | X | 4.2779-4.2549 | 4.2806-4.2609 |
| L-tryptophan |  |  |  | X | - | 7.5645-7.5404 |
| L-tyrosine | X | X | X | X | 6.9200-6.8860 | 6.9234-6.9080 |
| UDP-N-acetylglucosamine | X |  |  |  | 5.5321-5.5068 | - |
| UDP-N-acetylgalactosamine | X |  |  |  | 5.5690-5.5370 | - |
| UDP-sugar | X |  |  |  | 5.6080-5.5960 | - |
| uracil |  |  | X |  | 5.8226-5.8116 | - |
| uridine |  |  | X |  | 5.9286-5.9105 | - |
| L-valine | X | X | X | X | 1.0430-1.0361 | 1.0533-1.0475 |

| Lysates SKOV | AF | | | AuTM | | | CN1 | | | CN2 | | |
| --- | --- | --- | --- | --- | --- | --- | --- | --- | --- | --- | --- | --- |
|  | Log_2_(FC) | p-value | r | Log_2_(FC) | p-value | R | Log_2_(FC) | p-value | r | Log_2_(FC) | p-value | r |
| Valine | -0.101 | 0.028 | large | 0.024 | 0.342 | Negligible | 0.083 | 0.203 | negligible | 0.272 | 0.013 | large |
| Acetate | 0 | 0.226 | negligible | -0.057 | 0.256 | Negligible | 0.017 | 0.272 | negligible | -0.076 | 0.371 | negligible |
| Leucine | -0.076 | 0.030 | large | 0 | 0.377 | Medium | 0.076 | 0.222 | medium | 0.235 | 0.015 | large |
| Isoleucine | -0.082 | 0.046 | large | 0.034 | 0.307 | Negligible | 0.071 | 0.295 | medium | 0.274 | 0.019 | large |
| Alanine | -0.175 | 0.019 | large | -0.048 | 0.082 | Medium | 0.037 | 0.421 | negligible | 0.034 | 0.147 | large |
| Lysine | -0.002 | 0.295 | medium | -0.027 | 0.379 | Negligible | -0.046 | 0.445 | negligible | 0.217 | 0.020 | large |
| Proline | -0.115 | 0.070 | large | -0.12 | 0.079 | Large | 0.857 | 0.001 | large | 1.001 | 0.007 | large |
| Acetone | 0.438 | 0.087 | large | -0.279 | 0.314 | Negligible | 0.149 | 0.325 | negligible | 0.529 | 0.091 | large |
| Glutamate | -0.175 | 0.009 | large | -0.175 | 0.027 | Medium | -0.017 | 0.223 | negligible | -0.03 | 0.034 | medium |
| Succinate | -0.061 | 0.268 | medium | 1.854 | 0.006 | Large | -0.05 | 0.147 | negligible | -0.122 | 0.397 | negligible |
| Glutathione | 0.373 | 0.006 | large | 0.112 | 0.138 | Medium | -0.021 | 0.166 | negligible | -0.174 | 0.051 | large |
| Creatine | -0.256 | 0.035 | large | 0.216 | 0.120 | Medium | 0.003 | 0.244 | negligible | -0.316 | 0.011 | large |
| Choline | -0.048 | 0.256 | large | 0.026 | 0.366 | Negligible | 0.064 | 0.077 | medium | -0.004 | 0.235 | negligible |
| PC | 0.025 | 0.444 | negligible | 0.077 | 0.193 | Medium | 0.128 | 0.030 | large | 0.057 | 0.093 | large |
| GPC | 0.416 | 0.022 | large | 0.103 | 0.005 | Large | 0.079 | 0.018 | medium | 0.016 | 0.326 | negligible |
| Histidine | -0.074 | 0.326 | negligible | -0.071 | 0.424 | Negligible | -0.014 | 0.237 | negligible | 0.348 | 0.022 | large |
| Myo-inositol | -0.18 | 0.004 | large | -0.021 | 0.324 | Medium | -0.078 | 0.104 | large | -0.254 | 0.006 | large |
| Glycine | -0.265 | 0.013 | large | 0.068 | 0.198 | negligible | -0.619 | 0.010 | large | -0.417 | 0.027 | large |
| Threonine | -0.216 | 0.001 | large | -0.001 | 0.254 | medium | -0.066 | 0.109 | large | -0.001 | 0.301 | medium |
| D-Glucose | -0.09 | 0.096 | medium | -0.348 | 0.024 | large | -0.982 | 0.001 | large | -0.916 | 0.004 | large |
| Uridine | 0.752 | 0.031 | large | -0.117 | 0.311 | medium | -0.245 | 0.104 | large | -0.436 | 0.132 | medium |
| Fumarate | 0.067 | 0.359 | negligible | -0.379 | 0.010 | large | 0.112 | 0.132 | large | 0.053 | 0.353 | medium |
| Tyrosine | -0.073 | 0.160 | negligible | -0.109 | 0.383 | negligible | -0.029 | 0.356 | negligible | 0.309 | 0.023 | large |
| Phenylalanine | 0.049 | 0.487 | negligible | 0.122 | 0.185 | medium | 0.212 | 0.073 | large | 0.357 | 0.045 | large |
| Uracil | 0.655 | 0.002 | large | 0.267 | 0.054 | large | 0.017 | 0.338 | negligible | -0.038 | 0.128 | negligible |
| Inosine | 0.572 | 0.006 | large | 0.489 | 0.374 | negligible | 0.324 | 0.367 | negligible | -0.514 | 0.380 | negligible |
| ATP | -0.587 | 0.024 | large | -0.025 | 0.274 | negligible | 0.911 | 0.032 | large | 0.539 | 0.102 | large |
| AMP | -1.02 | 0.019 | large | -0.299 | 0.430 | medium | -0.046 | 0.402 | medium | 0.416 | 0.267 | medium |
| Niacinamide | -0.399 | 0.033 | large | -0.316 | 0.034 | large | 0.164 | 0.084 | large | 0.072 | 0.493 | negligible |
| 1-Methyl-nicotinamide | -0.286 | 0.050 | large | -0.025 | 0.268 | medium | 0.094 | 0.097 | large | 0.318 | 0.001 | large |

**Table S4**. List of metabolites analyzed in the cell lysates of SKOV-3 cells. For each comparison, the log2(FC), p-values and Cliff’s Delta magnitude ranking are reported.

**Table S5**. List of metabolites analyzed in the growth media of SKOV-3 cells. For each comparison, the log2(FC), p-values and Cliff’s Delta magnitude ranking are reported.

| Growth media SKOV | AF | | | AuTM | | | CN1 | | | CN2 | | |
| --- | --- | --- | --- | --- | --- | --- | --- | --- | --- | --- | --- | --- |
|  | Log_2_(FC) | p-value | r | Log_2_(FC) | p-value | r | Log_2_(FC) | p-value | r | Log_2_(FC) | p-value | r |
| Leucine | -0.009 | 0.124 | negligible | 0.006 | 0.047 | medium | -0.061 | 0.029 | large | -0.034 | 0.033 | large |
| Isoleucine | 0.000 | 0.378 | negligible | 0.016 | 0.004 | medium | -0.044 | 0.026 | large | -0.038 | 0.044 | large |
| Valine | 0.001 | 0.194 | negligible | 0.004 | 0.145 | negligible | -0.038 | 0.006 | large | -0.024 | 0.014 | large |
| 3-methyl-2-oxovalerate | 0.051 | 0.016 | large | 0.018 | 0.198 | negligible | 0.145 | 0.081 | large | 0.111 | 0.067 | large |
| Alanine | 0.043 | 0.001 | large | 0.021 | 0.345 | negligible | 0.032 | 0.141 | medium | -0.023 | 0.157 | medium |
| Acetate | -0.009 | 0.266 | negligible | -0.085 | 0.136 | large | -0.123 | 0.072 | large | -0.140 | 0.053 | large |
| Glutamate | 0.004 | 0.110 | medium | -0.170 | 0.015 | large | 0.009 | 0.428 | negligible | 0.034 | 0.012 | large |
| Pyruvate | 0.006 | 0.425 | negligible | -0.320 | 0.007 | large | -0.279 | 0.031 | large | -0.292 | 0.045 | large |
| Succinate | -0.003 | 0.379 | negligible | 0.305 | 0.002 | large | -0.067 | 0.029 | large | -0.066 | 0.009 | large |
| Citrate | -0.033 | 0.435 | medium | -0.292 | 0.061 | large | -0.115 | 0.325 | medium | -0.025 | 0.199 | negligible |
| Methionine | -0.336 | 0.022 | large | -0.426 | 0.017 | large | -0.166 | 0.091 | large | -0.013 | 0.173 | medium |
| Lysine | 0.017 | 0.116 | large | -0.010 | 0.076 | large | -0.034 | 0.084 | large | -0.011 | 0.016 | medium |
| Ornithine | 0.062 | 0.019 | large | 0.095 | 0.008 | large | 0.001 | 0.218 | medium | 0.018 | 0.295 | negligible |
| Choline | -0.013 | 0.191 | negligible | 0.007 | 0.232 | medium | -0.040 | 0.045 | large | 0.002 | 0.386 | negligible |
| D-Glucose | -0.033 | 0.009 | large | -0.005 | 0.090 | medium | -0.289 | 0.004 | large | -0.291 | 0.005 | large |
| Glycine | -0.053 | 0.012 | large | 0.020 | 0.006 | medium | -0.156 | 0.007 | large | -0.157 | 0.012 | large |
| Myo-inositol | 0.073 | 0.031 | large | -0.060 | 0.362 | negligible | 0.219 | 0.010 | large | 0.267 | 0.027 | large |
| Lactate | 0.157 | 0.010 | large | 0.094 | 0.037 | medium | 0.981 | 0.004 | large | 0.972 | 0.005 | large |
| Pyroglutamate | 0.080 | 0.057 | medium | 0.085 | 0.100 | negligible | 0.098 | 0.073 | large | 0.054 | 0.085 | medium |
| Threonine | 0.049 | 0.041 | large | 0.085 | 0.141 | large | 0.037 | 0.118 | large | -0.064 | 0.194 | medium |
| Fumarate | 0.398 | 0.206 | large | -0.116 | 0.322 | large | -0.219 | 0.449 | negligible | -0.113 | 0.350 | medium |
| Tyrosine | 0.009 | 0.220 | negligible | -0.014 | 0.178 | medium | -0.030 | 0.009 | large | -0.021 | 0.038 | large |
| Phenylalanine | -0.023 | 0.077 | large | 0.017 | 0.038 | large | -0.025 | 0.035 | large | -0.034 | 0.007 | large |
| Histidine | -0.014 | 0.450 | medium | -0.038 | 0.105 | large | 0.020 | 0.457 | medium | -0.002 | 0.484 | negligible |
| Formate | -0.020 | 0.406 | negligible | 0.061 | 0.028 | medium | -0.474 | 0.008 | large | -0.490 | 0.007 | large |
| Niacinamide | 0.050 | 0.297 | negligible | 0.236 | 0.410 | negligible | 0.115 | 0.236 | negligible | -0.284 | 0.182 | large |
| Tryptophan | 0.309 | 0.039 | large | 0.046 | 0.322 | medium | -0.032 | 0.142 | large | -0.012 | 0.367 | negligible |
| Serine | 0.041 | 0.282 | negligible | -0.058 | 0.303 | negligible | 0.194 | 0.032 | large | 0.185 | 0.020 | large |

| Lysates A2780 | AF | | | AuTM | | | CN1 | | | CN2 | | |
| --- | --- | --- | --- | --- | --- | --- | --- | --- | --- | --- | --- | --- |
|  | Log_2_(FC) | p-value | r | Log_2_(FC) | p-value | r | Log_2_(FC) | p-value | r | Log_2_(FC) | p-value | r |
| 4-hydroxyproline | -0.674 | 0.0002 | large | -0.233 | 0.036 | large | -0.347 | 0.037 | medium | -0.413 | 0.069 | large |
| Acetate | -0.215 | 0.0193 | large | -0.060 | 0.107 | medium | -0.054 | 0.022 | large | -0.256 | 0.053 | medium |
| Alanine | -0.487 | 0.0007 | large | -0.489 | 0.019 | large | -0.172 | 0.071 | medium | -0.223 | 0.014 | large |
| Asparagine | -0.619 | 0.0007 | large | -0.343 | 0.068 | medium | -0.522 | 0.001 | large | -0.970 | 0.014 | large |
| AXP | -0.443 | 0.003 | large | -0.184 | 0.034 | large | -0.109 | 0.408 | negligible | 0.070 | 0.783 | small |
| IMP | -0.191 | 0.010 | large | -0.134 | 0.031 | large | -0.060 | 0.797 | negligible | -0.125 | 0.107 | large |
| Choline | 0.066 | 0.121 | medium | -0.008 | 0.970 | negligible | 0.222 | 0.007 | large | 0.429 | 0.106 | large |
| Citrate | -0.051 | 0.397 | small | -0.226 | 0.048 | large | -0.074 | 0.600 | large | -0.490 | 0.077 | large |
| Creatine | -0.334 | 0.000001 | large | -0.077 | 0.036 | small | -0.036 | 0.470 | negligible | -0.049 | 0.150 | medium |
| Fumarate | -0.503 | 0.0013 | large | -0.327 | 0.012 | large | 0.122 | 0.623 | medium | 0.280 | 0.083 | medium |
| Glucose-1-phosphate | 0.001 | 0.434 | negligible | -0.844 | 0.071 | large | 0.873 | 0.026 | large | 2.039 | 0.0002 | large |
| Glutamate | -0.312 | 0.00002 | large | -0.189 | 0.023 | large | -0.158 | 0.017 | large | -0.389 | 0.001 | large |
| Glutamine | 0.457 | 0.002 | large | -0.012 | 0.784 | negligible | -0.363 | 0.034 | large | -2.099 | 0.002 | large |
| Glutathione | 1.432 | 0.0001 | large | 0.184 | 0.105 | small | -0.119 | 0.532 | negligible | -0.652 | 0.003 | large |
| Glycine | -0.190 | 0.002 | large | -0.144 | 0.018 | large | -0.205 | 0.021 | large | 0.214 | 0.209 | large |
| GPC | 0.084 | 0.204 | small | 0.084 | 0.289 | medium | -0.114 | 0.665 | small | -0.114 | 0.217 | large |
| GXP | -0.302 | 0.087 | large | -0.190 | 0.069 | medium | 0.163 | 0.153 | small | 0.003 | 0.926 | negligible |
| Histidine | 0.667 | 0.0001 | large | -0.204 | 0.096 | large | -0.210 | 0.376 | medium | 0.242 | 0.256 | medium |
| Isoleucine | -0.495 | 0.014 | large | -0.208 | 0.026 | large | -0.060 | 0.365 | negligible | 0.070 | 0.367 | negligible |
| Lactate | -1.082 | 0.009 | large | -0.912 | 0.237 | small | 0.670 | 0.010 | large | 1.389 | 0.002 | large |
| Leucine | -0.151 | 0.026 | large | -0.061 | 0.149 | medium | 0.011 | 0.770 | negligible | 0.123 | 0.017 | large |
| Myo-inositol | -0.265 | 0.004 | large | -0.112 | 0.432 | large | -0.100 | 0.009 | medium | -0.237 | 0.006 | large |
| Niacinamide | -0.213 | 0.243 | large | -0.053 | 0.837 | negligible | -0.277 | 0.073 | medium | -0.002 | 0.581 | negligible |
| PC | 0.807 | 0.000001 | large | -0.038 | 0.349 | negligible | 0.115 | 0.042 | large | -0.204 | 0.029 | large |
| Phenylalanine | 0.472 | 0.007 | large | 0.208 | 0.003 | medium | 0.410 | 0.006 | large | 0.743 | 0.057 | large |
| Succinate | -0.244 | 0.008 | large | 0.887 | 0.001 | large | -0.026 | 0.592 | small | -0.295 | 0.167 | large |
| Taurine | -0.140 | 0.0318 | medium | 0.071 | 0.055602 | small | 0.039 | 0.023 | medium | -0.074 | 0.067 | medium |
| Tyrosine | 0.007 | 0.5933 | negligible | -0.231 | 0.424297 | small | -0.022 | 0.946 | negligible | -0.013 | 0.792 | negligible |
| UDP-N-acetylglucosamine | 0.786 | 0.0008 | large | -0.285 | 0.170095 | small | -0.663 | 0.0005 | large | -1.386 | 0.00001 | large |
| UDP-N-galactosamine | 0.566 | 0.0117 | large | -0.122 | 0.471082 | small | -1.142 | 0.004 | large | -1.072 | 0.014 | large |
| UDP-sugar | -0.165 | 0.6000 | medium | -0.457 | 0.08484 | large | 0.206 | 0.994 | negligible | -0.540 | 0.084 | large |
| Valine | 0.211 | 0.0091 | medium | -0.033 | 0.688193 | negligible | 0.131 | 0.094 | large | 0.432 | 0.001 | large |

**Table S6**. List of metabolites analyzed in the cell lysates of A2780 cells. For each comparison, the log2(FC), p-values and Cliff’s Delta magnitude ranking are reported.

**Table S7**. List of metabolites analyzed in the growth media of A2780 cells. For each comparison, the log2(FC), p-values and Cliff’s Delta magnitude ranking are reported.

| Growth media A2780 | AF | | | AuTM | | | CN1 | | | CN2 | | |
| --- | --- | --- | --- | --- | --- | --- | --- | --- | --- | --- | --- | --- |
|  | Log_2_(FC) | p-value | r | Log_2_(FC) | p-value | r | Log_2_(FC) | p-value | r | Log_2_(FC) | p-value | r |
| 3-hydroxyisovalerate | 0.050 | 0.305 | negligible | 0.110 | 0.122 | medium | 0.699 | 0.004 | large | 0.338 | 0.152 | small |
| 3-methyl-2-oxovalerate | -0.031 | 0.073 | small | -0.226 | 0.062 | medium | -0.317 | 0.00002 | large | -0.107 | 0.001 | large |
| Acetate | 0.030 | 0.002 | large | -0.070 | 0.002 | large | -0.097 | 0.141 | large | -0.149 | 0.00001 | large |
| Acetone | -0.099 | 0.092 | large | 0.011 | 0.667 | small | -0.034 | 0.373 | small | -0.004 | 0.969 | negligible |
| Alanine | 0.068 | 0.004 | large | -0.274 | 0.009 | large | 0.241 | 0.002 | large | 0.168 | 0.120 | large |
| Arginine | 0.072 | 0.002 | large | 0.005 | 0.294 | negligible | 0.008 | 0.380 | small | 0.022 | 0.242 | small |
| Asparagine | 0.063 | 0.528 | medium | -0.031 | 0.030 | large | 0.008 | 0.655 | negligible | -0.069 | 0.072 | large |
| Aspartate | -0.055 | 0.298 | medium | -0.016 | 0.295 | small | 0.077 | 0.334 | medium | -0.056 | 0.731 | small |
| Choline | -0.138 | 0.045 | small | 0.043 | 0.410 | small | 0.577 | 0.0001 | large | 1.421 | 0.0002 | large |
| Citrate | -0.036 | 0.113 | medium | 0.045 | 0.401 | small | -0.169 | 0.130 | large | -0.168 | 0.003 | large |
| Cystine | -0.314 | 0.004 | large | -0.166 | 0.001 | large | 0.123 | 0.011 | large | 0.213 | 0.001 | large |
| Formate | 0.246 | 0.017 | large | -0.119 | 0.108 | large | -0.066 | 0.134 | large | -0.388 | 0.005 | large |
| Fumarate | 0.022 | 0.715 | small | -0.163 | 0.211 | small | -0.441 | 0.430 | large | -0.297 | 0.074 | medium |
| D-Glucose | 0.225 | 0.00001 | large | 0.068 | 0.010 | medium | -0.506 | 0.0002 | large | -1.260 | 0.0001 | large |
| Glutamate | 0.065 | 0.029 | large | -0.063 | 0.002 | large | -0.069 | 0.0001 | large | -0.151 | 0.0007 | large |
| Glutamine | 0.486 | 0.000001 | large | 0.033 | 0.258 | small | 0.305 | 0.000002 | large | 0.724 | 0.00001 | large |
| Glycine | 0.083 | 0.005 | large | -0.024 | 0.153 | small | -0.057 | 0.075 | large | -0.233 | 0.001 | large |
| Histidine | 0.240 | 0.002 | large | 0.006 | 0.767 | negligible | 0.130 | 0.063 | large | 0.286 | 0.006 | large |
| Isoleucine | 0.208 | 0.000002 | large | 0.224 | 0.002 | large | 0.217 | 0.0003 | large | 0.244 | 0.0003 | large |
| Lactate | -0.135 | 0.00001 | large | -0.041 | 0.051 | medium | 0.173 | 0.0002 | large | 0.359 | 0.0002 | large |
| Leucine | 0.324 | 0.00000001 | large | 0.204 | 0.001 | large | 0.218 | 0.00003 | large | 0.336 | 0.0004 | large |
| Lysine | 0.505 | 0.00008 | large | 0.033 | 0.433 | small | 0.255 | 0.001 | large | 0.454 | 0.001 | large |
| Methionine | 0.236 | 0.432 | large | -0.137 | 0.124 | large | #NUM! | 0.387 | small | 0.347 | 0.005 | large |
| Myo-inositol | -0.045 | 0.007 | large | 0.000 | 0.616 | small | -0.055 | 0.009 | large | -0.110 | 0.001 | large |
| Ornithine | 0.046 | 0.863 | small | 0.017 | 0.205 | small | -0.028 | 0.435 | negligible | -0.026 | 0.293 | negligible |
| Phenylalanine | 0.779 | 0.00003 | large | 0.106 | 0.090 | small | 0.434 | 0.004 | large | 0.706 | 0.002 | large |
| Proline | 0.021 | 0.785 | small | 0.090 | 0.332 | medium | 0.058 | 0.115 | medium | 0.107 | 0.058 | large |
| Pyroglutamate | 0.218 | 0.0003 | large | 0.025 | 0.178 | small | -0.090 | 0.229 | small | -0.271 | 0.001 | large |
| Pyruvate | -0.118 | 0.0001 | large | -0.649 | 0.0005 | large | -0.488 | 0.0003 | large | -0.630 | 0.001 | large |
| Serine | 0.125 | 0.390 | small | -0.133 | 0.170 | small | 0.015 | 0.776 | negligible | 0.337 | 0.004 | large |
| Succinate | 0.020 | 0.495 | negligible | 0.137 | 0.002 | large | -0.222 | 0.021 | large | -0.202 | 0.005 | large |
| Tyrosine | 0.533 | 0.0001 | large | 0.107 | 0.029 | medium | 0.293 | 0.021 | large | 0.489 | 0.004 | large |
| Valine | 1.109 | 0.00000003 | large | 0.270 | 0.001 | large | 0.458 | 0.000001 | large | 0.776 | 0.0004 | large |

**Table S8**. Pathway enrichment output generated by MetaboAnalyst for AF treatment in SKOV-3 cells.

|  | Total | Expected | Hits |  | p-value | log10(p-value) | Holm adjust | FDR | Impact |
| --- | --- | --- | --- | --- | --- | --- | --- | --- | --- |
| Valine, leucine and isoleucine biosynthesis | 8 | 0.1206 | 5 |  | 2.73E-08 | 7.5643 | 2.18E-06 | 2.18E-06 | 0.75 |
| Glutathione metabolism | 28 | 0.42211 | 4 |  | 0.00063975 | 3.194 | 0.05054 | 0.02559 | 0.24325 |
| Valine, leucine and isoleucine degradation | 40 | 0.60302 | 4 |  | 0.0025281 | 2.5972 | 0.19719 | 0.067416 | 0.07548 |
| Glycolysis or Gluconeogenesis | 26 | 0.39196 | 3 |  | 0.0062377 | 2.205 | 0.4803 | 0.099803 | 0.11764 |
| One carbon pool by folate | 26 | 0.39196 | 3 |  | 0.0062377 | 2.205 | 0.4803 | 0.099803 | 0.12963 |
| Glycine, serine and threonine metabolism | 33 | 0.49749 | 3 |  | 0.012212 | 1.9132 | 0.91593 | 0.16283 | 0.2439 |
| Arginine and proline metabolism | 36 | 0.54271 | 3 |  | 0.015514 | 1.8093 | 1 | 0.17731 | 0.10527 |
| Arginine biosynthesis | 14 | 0.21106 | 2 |  | 0.017753 | 1.7507 | 1 | 0.17753 | 0.17647 |
| Pantothenate and CoA biosynthesis | 20 | 0.30151 | 2 |  | 0.035082 | 1.4549 | 1 | 0.31184 | 0.05263 |
| Galactose metabolism | 27 | 0.40704 | 2 |  | 0.060799 | 1.2161 | 1 | 0.4653 | 0.03125 |
| Alanine, aspartate and glutamate metabolism | 28 | 0.42211 | 2 |  | 0.064883 | 1.1879 | 1 | 0.4653 | 0.15625 |
| Porphyrin metabolism | 31 | 0.46734 | 2 |  | 0.077672 | 1.1097 | 1 | 0.4653 | 0.07408 |
| Glyoxylate and dicarboxylate metabolism | 32 | 0.48241 | 2 |  | 0.082103 | 1.0856 | 1 | 0.4653 | 0.1 |
| Purine metabolism | 70 | 1.0553 | 3 |  | 0.085109 | 1.07 | 1 | 0.4653 | 0.08989 |
| Nitrogen metabolism | 6 | 0.090452 | 1 |  | 0.087243 | 1.0593 | 1 | 0.4653 | 0.25 |
| Pyrimidine metabolism | 39 | 0.58794 | 2 |  | 0.11514 | 0.93876 | 1 | 0.57571 | 0.03509 |
| Ascorbate and aldarate metabolism | 9 | 0.13568 | 1 |  | 0.12808 | 0.89251 | 1 | 0.60273 | 0.125 |
| Nicotinate and nicotinamide metabolism | 15 | 0.22613 | 1 |  | 0.20457 | 0.68917 | 1 | 0.86133 | 0.04762 |
| Butanoate metabolism | 15 | 0.22613 | 1 |  | 0.20457 | 0.68917 | 1 | 0.86133 | 0.06667 |
| Histidine metabolism | 16 | 0.24121 | 1 |  | 0.21667 | 0.6642 | 1 | 0.86669 | 0.06667 |
| Fructose and mannose metabolism | 20 | 0.30151 | 1 |  | 0.26335 | 0.57946 | 1 | 0.91533 | 0 |
| Selenocompound metabolism | 20 | 0.30151 | 1 |  | 0.26335 | 0.57946 | 1 | 0.91533 | 0 |
| Ether lipid metabolism | 20 | 0.30151 | 1 |  | 0.26335 | 0.57946 | 1 | 0.91533 | 0 |
| beta-Alanine metabolism | 21 | 0.31658 | 1 |  | 0.2746 | 0.5613 | 1 | 0.91533 | 0 |
| Pyruvate metabolism | 23 | 0.34673 | 1 |  | 0.2966 | 0.52783 | 1 | 0.94912 | 0.03571 |
| Lipoic acid metabolism | 28 | 0.42211 | 1 |  | 0.34884 | 0.45737 | 1 | 1 | 0.04878 |
| Inositol phosphate metabolism | 30 | 0.45226 | 1 |  | 0.36868 | 0.43335 | 1 | 1 | 0.05 |
| Cysteine and methionine metabolism | 33 | 0.49749 | 1 |  | 0.39735 | 0.40082 | 1 | 1 | 0.0303 |
| Glycerophospholipid metabolism | 36 | 0.54271 | 1 |  | 0.42478 | 0.37184 | 1 | 1 | 0.03774 |
| Tryptophan metabolism | 41 | 0.61809 | 1 |  | 0.46784 | 0.3299 | 1 | 1 | 0.10256 |
| Amino sugar and nucleotide sugar metabolism | 42 | 0.63317 | 1 |  | 0.47608 | 0.32232 | 1 | 1 | 0.02326 |
| Primary bile acid biosynthesis | 46 | 0.69347 | 1 |  | 0.50781 | 0.2943 | 1 | 1 | 0.04082 |

**Table S9**. Pathway enrichment output generated by MetaboAnalyst for AuTM treatment in SKOV-3 cells.

|  | Total | Expected | Hits | p-value | log10(p-value) | Holm adjust | FDR | Impact |
| --- | --- | --- | --- | --- | --- | --- | --- | --- |
| Glycolysis or Gluconeogenesis | 26 | 0.26131 | 4 | 8.93E-05 | 4.0493 | 0.0071414 | 0.004831 | 0.14705 |
| Alanine, aspartate and glutamate metabolism | 28 | 0.28141 | 4 | 0.000121 | 3.918 | 0.0095412 | 0.004831 | 0.125 |
| Glyoxylate and dicarboxylate metabolism | 32 | 0.32161 | 4 | 0.000207 | 3.684 | 0.016146 | 0.00552 | 0.1 |
| Arginine biosynthesis | 14 | 0.1407 | 3 | 0.000284 | 3.547 | 0.021853 | 0.005676 | 0.17647 |
| Citrate cycle (TCA cycle) | 20 | 0.20101 | 3 | 0.000857 | 3.0672 | 0.065099 | 0.013705 | 0.13794 |
| Pyruvate metabolism | 23 | 0.23116 | 3 | 0.001306 | 2.884 | 0.097973 | 0.017417 | 0.10714 |
| Glutathione metabolism | 28 | 0.28141 | 3 | 0.002343 | 2.6302 | 0.17339 | 0.025611 | 0.08109 |
| Valine, leucine and isoleucine biosynthesis | 8 | 0.080402 | 2 | 0.002561 | 2.5916 | 0.18696 | 0.025611 | 0.25 |
| Arginine and proline metabolism | 36 | 0.36181 | 3 | 0.004861 | 2.3133 | 0.34998 | 0.043207 | 0.07895 |
| Butanoate metabolism | 15 | 0.15075 | 2 | 0.009217 | 2.0354 | 0.65443 | 0.073738 | 0.06667 |
| One carbon pool by folate | 26 | 0.26131 | 2 | 0.02675 | 1.5727 | 1 | 0.19454 | 0.11111 |
| Lipoic acid metabolism | 28 | 0.28141 | 2 | 0.03075 | 1.5122 | 1 | 0.205 | 0.07317 |
| Porphyrin metabolism | 31 | 0.31156 | 2 | 0.037171 | 1.4298 | 1 | 0.20859 | 0.07408 |
| Phenylalanine, tyrosine and tryptophan biosynthesis | 4 | 0.040201 | 1 | 0.039636 | 1.4019 | 1 | 0.20859 | 0.5 |
| Cysteine and methionine metabolism | 33 | 0.33166 | 2 | 0.041717 | 1.3797 | 1 | 0.20859 | 0.0303 |
| Glycine, serine and threonine metabolism | 33 | 0.33166 | 2 | 0.041717 | 1.3797 | 1 | 0.20859 | 0.21951 |
| Nitrogen metabolism | 6 | 0.060302 | 1 | 0.058897 | 1.2299 | 1 | 0.26295 | 0.25 |
| Valine, leucine and isoleucine degradation | 40 | 0.40201 | 2 | 0.059164 | 1.2279 | 1 | 0.26295 | 0.03774 |
| Tyrosine metabolism | 42 | 0.42211 | 2 | 0.064553 | 1.1901 | 1 | 0.2718 | 0 |
| Phenylalanine metabolism | 8 | 0.080402 | 1 | 0.077795 | 1.109 | 1 | 0.31118 | 0.375 |
| Nicotinate and nicotinamide metabolism | 15 | 0.15075 | 1 | 0.14118 | 0.85024 | 1 | 0.53781 | 0.04762 |
| Histidine metabolism | 16 | 0.1608 | 1 | 0.14989 | 0.82423 | 1 | 0.54505 | 0.06667 |
| Fructose and mannose metabolism | 20 | 0.20101 | 1 | 0.18392 | 0.73537 | 1 | 0.61307 | 0 |
| Ether lipid metabolism | 20 | 0.20101 | 1 | 0.18392 | 0.73537 | 1 | 0.61307 | 0 |
| Propanoate metabolism | 22 | 0.22111 | 1 | 0.20045 | 0.69799 | 1 | 0.64145 | 0.04 |
| Galactose metabolism | 27 | 0.27136 | 1 | 0.24042 | 0.61902 | 1 | 0.73976 | 0.03125 |
| Glycerophospholipid metabolism | 36 | 0.36181 | 1 | 0.30769 | 0.51189 | 1 | 0.91167 | 0.03774 |
| Amino sugar and nucleotide sugar metabolism | 42 | 0.42211 | 1 | 0.34938 | 0.4567 | 1 | 0.99824 | 0.02326 |
| Primary bile acid biosynthesis | 46 | 0.46231 | 1 | 0.37586 | 0.42497 | 1 | 1 | 0.04082 |

**Table S10**. Pathway enrichment output generated by MetaboAnalyst for CN1 treatment in SKOV-3 cells.

|  | Total | Expected | Hits | p-value | log10(p-value) | Holm adjust | FDR | Impact |
| --- | --- | --- | --- | --- | --- | --- | --- | --- |
| Valine, leucine and isoleucine biosynthesis | 8 | 0.095477 | 3 | 7.78E-05 | 4.1088 | 0.006227 | 0.004903 | 0.25 |
| Glycolysis or Gluconeogenesis | 26 | 0.3103 | 4 | 0.000184 | 3.7355 | 0.014524 | 0.004903 | 0.14705 |
| One carbon pool by folate | 26 | 0.3103 | 4 | 0.000184 | 3.7355 | 0.014524 | 0.004903 | 0.14815 |
| Glyoxylate and dicarboxylate metabolism | 32 | 0.38191 | 4 | 0.000422 | 3.3742 | 0.03253 | 0.007633 | 0.13334 |
| Glycine, serine and threonine metabolism | 33 | 0.39384 | 4 | 0.000477 | 3.3214 | 0.036258 | 0.007633 | 0.39024 |
| Phenylalanine, tyrosine and tryptophan biosynthesis | 4 | 0.047739 | 2 | 0.000799 | 3.0976 | 0.059898 | 0.010649 | 0.75 |
| Phenylalanine metabolism | 8 | 0.095477 | 2 | 0.003622 | 2.441 | 0.26803 | 0.041395 | 0.375 |
| Glycerophospholipid metabolism | 36 | 0.42965 | 3 | 0.008027 | 2.0954 | 0.58598 | 0.080271 | 0.11322 |
| Valine, leucine and isoleucine degradation | 40 | 0.47739 | 3 | 0.010776 | 1.9675 | 0.77588 | 0.095788 | 0.05661 |
| Citrate cycle (TCA cycle) | 20 | 0.23869 | 2 | 0.022566 | 1.6465 | 1 | 0.18053 | 0.13794 |
| Pyruvate metabolism | 23 | 0.2745 | 2 | 0.029416 | 1.5314 | 1 | 0.21393 | 0.10714 |
| Galactose metabolism | 27 | 0.32224 | 2 | 0.039671 | 1.4015 | 1 | 0.24241 | 0.03125 |
| Alanine, aspartate and glutamate metabolism | 28 | 0.33417 | 2 | 0.042422 | 1.3724 | 1 | 0.24241 | 0 |
| Lipoic acid metabolism | 28 | 0.33417 | 2 | 0.042422 | 1.3724 | 1 | 0.24241 | 0.07317 |
| Cysteine and methionine metabolism | 33 | 0.39384 | 2 | 0.0572 | 1.2426 | 1 | 0.30507 | 0.06061 |
| Arginine and proline metabolism | 36 | 0.42965 | 2 | 0.066823 | 1.1751 | 1 | 0.33412 | 0.05263 |
| Tyrosine metabolism | 42 | 0.50126 | 2 | 0.087554 | 1.0577 | 1 | 0.41202 | 0.11628 |
| Ascorbate and aldarate metabolism | 9 | 0.10741 | 1 | 0.10267 | 0.98855 | 1 | 0.45631 | 0.125 |
| Butanoate metabolism | 15 | 0.17902 | 1 | 0.16548 | 0.78125 | 1 | 0.66192 | 0 |
| D-Amino acid metabolism | 15 | 0.17902 | 1 | 0.16548 | 0.78125 | 1 | 0.66192 | 0.125 |
| Ubiquinone and other terpenoid-quinone biosynthesis | 19 | 0.22676 | 1 | 0.20501 | 0.68822 | 1 | 0.71539 | 0.0625 |
| Fructose and mannose metabolism | 20 | 0.23869 | 1 | 0.21462 | 0.66834 | 1 | 0.71539 | 0 |
| Ether lipid metabolism | 20 | 0.23869 | 1 | 0.21462 | 0.66834 | 1 | 0.71539 | 0 |
| Pantothenate and CoA biosynthesis | 20 | 0.23869 | 1 | 0.21462 | 0.66834 | 1 | 0.71539 | 0.05263 |
| Propanoate metabolism | 22 | 0.26256 | 1 | 0.23349 | 0.63173 | 1 | 0.74718 | 0.04 |
| Glutathione metabolism | 28 | 0.33417 | 1 | 0.28758 | 0.54125 | 1 | 0.88485 | 0.02703 |
| Inositol phosphate metabolism | 30 | 0.35804 | 1 | 0.30479 | 0.516 | 1 | 0.88718 | 0.05 |
| Porphyrin metabolism | 31 | 0.36997 | 1 | 0.31324 | 0.50412 | 1 | 0.88718 | 0.03704 |
| Sphingolipid metabolism | 32 | 0.38191 | 1 | 0.3216 | 0.49268 | 1 | 0.88718 | 0.02174 |
| Amino sugar and nucleotide sugar metabolism | 42 | 0.50126 | 1 | 0.40006 | 0.39788 | 1 | 1 | 0.02326 |
| Primary bile acid biosynthesis | 46 | 0.54899 | 1 | 0.42896 | 0.36758 | 1 | 1 | 0.04082 |
| Purine metabolism | 70 | 0.83543 | 1 | 0.57655 | 0.23916 | 1 | 1 | 0.02247 |

**Table S11**. Pathway enrichment output generated by MetaboAnalyst for CN2 treatment in SKOV-3 cells.

|  | Total | Expected | Hits | p-value | log10(p-value) | Holm adjust | FDR | Impact |
| --- | --- | --- | --- | --- | --- | --- | --- | --- |
| Glyoxylate and dicarboxylate metabolism | 32 | 0.40201 | 5 | 2.98E-05 | 4.5265 | 0.00238 | 0.00238 | 0.16667 |
| Valine, leucine and isoleucine biosynthesis | 8 | 0.1005 | 3 | 9.14E-05 | 4.0393 | 0.007217 | 0.003654 | 0.25 |
| Glycolysis or Gluconeogenesis | 26 | 0.32663 | 4 | 0.000227 | 3.6435 | 0.017727 | 0.006061 | 0.14705 |
| Glycine, serine and threonine metabolism | 33 | 0.41457 | 4 | 0.000588 | 3.2309 | 0.04525 | 0.011753 | 0.36585 |
| Arginine and proline metabolism | 36 | 0.45226 | 4 | 0.000826 | 3.0833 | 0.062742 | 0.011822 | 0.10527 |
| Phenylalanine, tyrosine and tryptophan biosynthesis | 4 | 0.050251 | 2 | 0.000887 | 3.0523 | 0.066497 | 0.011822 | 0.75 |
| Phenylalanine metabolism | 8 | 0.1005 | 2 | 0.004014 | 2.3964 | 0.29706 | 0.045496 | 0.375 |
| Alanine, aspartate and glutamate metabolism | 28 | 0.35176 | 3 | 0.00455 | 2.342 | 0.33212 | 0.045496 | 0.125 |
| Valine, leucine and isoleucine degradation | 40 | 0.50251 | 3 | 0.012459 | 1.9045 | 0.89705 | 0.11075 | 0.05661 |
| Butanoate metabolism | 15 | 0.18844 | 2 | 0.01428 | 1.8453 | 1 | 0.11424 | 0.06667 |
| Histidine metabolism | 16 | 0.20101 | 2 | 0.016197 | 1.7906 | 1 | 0.1178 | 0.26667 |
| Citrate cycle (TCA cycle) | 20 | 0.25126 | 2 | 0.024886 | 1.604 | 1 | 0.16591 | 0.13794 |
| Pyruvate metabolism | 23 | 0.28894 | 2 | 0.0324 | 1.4895 | 1 | 0.19938 | 0.10714 |
| One carbon pool by folate | 26 | 0.32663 | 2 | 0.040696 | 1.3905 | 1 | 0.21943 | 0.11111 |
| Galactose metabolism | 27 | 0.3392 | 2 | 0.043624 | 1.3603 | 1 | 0.21943 | 0.03125 |
| Glutathione metabolism | 28 | 0.35176 | 2 | 0.04663 | 1.3313 | 1 | 0.21943 | 0.05406 |
| Lipoic acid metabolism | 28 | 0.35176 | 2 | 0.04663 | 1.3313 | 1 | 0.21943 | 0.07317 |
| Porphyrin metabolism | 31 | 0.38945 | 2 | 0.056091 | 1.2511 | 1 | 0.24929 | 0.07408 |
| Cysteine and methionine metabolism | 33 | 0.41457 | 2 | 0.062747 | 1.2024 | 1 | 0.2642 | 0.06061 |
| Nitrogen metabolism | 6 | 0.075377 | 1 | 0.07316 | 1.1357 | 1 | 0.29264 | 0.25 |
| Tyrosine metabolism | 42 | 0.52764 | 2 | 0.095699 | 1.0191 | 1 | 0.36457 | 0.11628 |
| Ascorbate and aldarate metabolism | 9 | 0.11307 | 1 | 0.1078 | 0.96736 | 1 | 0.39202 | 0.125 |
| Biotin metabolism | 10 | 0.12563 | 1 | 0.11908 | 0.92417 | 1 | 0.41418 | 0 |
| Arginine biosynthesis | 14 | 0.17588 | 1 | 0.16283 | 0.78827 | 1 | 0.53365 | 0.11765 |
| Nicotinate and nicotinamide metabolism | 15 | 0.18844 | 1 | 0.17344 | 0.76086 | 1 | 0.53365 | 0.09524 |
| D-Amino acid metabolism | 15 | 0.18844 | 1 | 0.17344 | 0.76086 | 1 | 0.53365 | 0.125 |
| Ubiquinone and other terpenoid-quinone biosynthesis | 19 | 0.23869 | 1 | 0.21462 | 0.66834 | 1 | 0.61959 | 0.0625 |
| Fructose and mannose metabolism | 20 | 0.25126 | 1 | 0.2246 | 0.64859 | 1 | 0.61959 | 0 |
| Pantothenate and CoA biosynthesis | 20 | 0.25126 | 1 | 0.2246 | 0.64859 | 1 | 0.61959 | 0.05263 |
| beta-Alanine metabolism | 21 | 0.26382 | 1 | 0.23447 | 0.62992 | 1 | 0.62525 | 0 |
| Propanoate metabolism | 22 | 0.27638 | 1 | 0.24421 | 0.61223 | 1 | 0.63023 | 0.04 |
| Lysine degradation | 30 | 0.37688 | 1 | 0.31805 | 0.49751 | 1 | 0.77102 | 0.03571 |
| Inositol phosphate metabolism | 30 | 0.37688 | 1 | 0.31805 | 0.49751 | 1 | 0.77102 | 0.05 |
| Sphingolipid metabolism | 32 | 0.40201 | 1 | 0.3354 | 0.47443 | 1 | 0.78918 | 0.02174 |
| Amino sugar and nucleotide sugar metabolism | 42 | 0.52764 | 1 | 0.41608 | 0.38083 | 1 | 0.95103 | 0.02326 |
| Primary bile acid biosynthesis | 46 | 0.57789 | 1 | 0.44566 | 0.35099 | 1 | 0.99036 | 0.04082 |

**Table S12**. Pathway enrichment output generated by MetaboAnalyst for AF treatment in A2780 cells.

|  | Total | Expected | Hits | p-value | log10(p-value) | Holm adjust | FDR | Impact |
| --- | --- | --- | --- | --- | --- | --- | --- | --- |
| Alanine, aspartate and glutamate metabolism | 28 | 0.56281 | 8 | 2.48E-08 | 7.6062 | 1.98E-06 | 1.98E-06 | 0.375 |
| Glyoxylate and dicarboxylate metabolism | 32 | 0.64322 | 7 | 1.57E-06 | 5.8027 | 0.000124 | 6.30E-05 | 0.13333 |
| Glycolysis or Gluconeogenesis | 26 | 0.52261 | 5 | 0.000116 | 3.9361 | 0.009037 | 0.002343 | 0.17646 |
| Arginine biosynthesis | 14 | 0.28141 | 4 | 0.000117 | 3.9313 | 0.009037 | 0.002343 | 0.41177 |
| Valine, leucine and isoleucine biosynthesis | 8 | 0.1608 | 3 | 0.000386 | 3.4131 | 0.029358 | 0.006181 | 0.25 |
| Citrate cycle (TCA cycle) | 20 | 0.40201 | 4 | 0.000521 | 3.2835 | 0.039048 | 0.006576 | 0.17242 |
| Arginine and proline metabolism | 36 | 0.72362 | 5 | 0.000575 | 3.24 | 0.04258 | 0.006576 | 0.18422 |
| Pyruvate metabolism | 23 | 0.46231 | 4 | 0.000912 | 3.04 | 0.06657 | 0.009119 | 0.14285 |
| Glutathione metabolism | 28 | 0.56281 | 4 | 0.001964 | 2.7068 | 0.14143 | 0.017461 | 0.24325 |
| Phenylalanine, tyrosine and tryptophan biosynthesis | 4 | 0.080402 | 2 | 0.002291 | 2.6399 | 0.16267 | 0.01833 | 0.75 |
| Glycine, serine and threonine metabolism | 33 | 0.66332 | 4 | 0.003657 | 2.4368 | 0.25601 | 0.026599 | 0.2439 |
| Nitrogen metabolism | 6 | 0.1206 | 2 | 0.005585 | 2.253 | 0.38538 | 0.037235 | 0.25 |
| Phenylalanine metabolism | 8 | 0.1608 | 2 | 0.010166 | 1.9928 | 0.69131 | 0.062562 | 0.375 |
| Butanoate metabolism | 15 | 0.30151 | 2 | 0.034923 | 1.4569 | 1 | 0.19956 | 0.06667 |
| Histidine metabolism | 16 | 0.32161 | 2 | 0.039418 | 1.4043 | 1 | 0.21023 | 0.26667 |
| Valine, leucine and isoleucine degradation | 40 | 0.80402 | 3 | 0.044039 | 1.3562 | 1 | 0.22019 | 0.05661 |
| Amino sugar and nucleotide sugar metabolism | 42 | 0.84422 | 3 | 0.049806 | 1.3027 | 1 | 0.22136 | 0.04652 |
| Tyrosine metabolism | 42 | 0.84422 | 3 | 0.049806 | 1.3027 | 1 | 0.22136 | 0.11628 |
| One carbon pool by folate | 26 | 0.52261 | 2 | 0.094327 | 1.0254 | 1 | 0.39717 | 0.11111 |
| Galactose metabolism | 27 | 0.54271 | 2 | 0.10063 | 0.99728 | 1 | 0.40252 | 0.03125 |
| Lipoic acid metabolism | 28 | 0.56281 | 2 | 0.10705 | 0.97042 | 1 | 0.4078 | 0.07317 |
| Porphyrin metabolism | 31 | 0.62312 | 2 | 0.12694 | 0.89641 | 1 | 0.46159 | 0.07408 |
| Taurine and hypotaurine metabolism | 8 | 0.1608 | 1 | 0.15024 | 0.82321 | 1 | 0.52258 | 0.28571 |
| Ascorbate and aldarate metabolism | 9 | 0.1809 | 1 | 0.16741 | 0.77622 | 1 | 0.55803 | 0.125 |
| Pyrimidine metabolism | 39 | 0.78392 | 2 | 0.18355 | 0.73625 | 1 | 0.56689 | 0.05263 |
| Biotin metabolism | 10 | 0.20101 | 1 | 0.18424 | 0.73462 | 1 | 0.56689 | 0 |
| Primary bile acid biosynthesis | 46 | 0.92462 | 2 | 0.23569 | 0.62765 | 1 | 0.69835 | 0.08164 |
| Ubiquinone and other terpenoid-quinone biosynthesis | 19 | 0.38191 | 1 | 0.3216 | 0.49268 | 1 | 0.86556 | 0.0625 |
| Fructose and mannose metabolism | 20 | 0.40201 | 1 | 0.3354 | 0.47443 | 1 | 0.86556 | 0 |
| Selenocompound metabolism | 20 | 0.40201 | 1 | 0.3354 | 0.47443 | 1 | 0.86556 | 0 |
| Pantothenate and CoA biosynthesis | 20 | 0.40201 | 1 | 0.3354 | 0.47443 | 1 | 0.86556 | 0.05263 |
| beta-Alanine metabolism | 21 | 0.42211 | 1 | 0.34893 | 0.45726 | 1 | 0.87233 | 0 |
| Propanoate metabolism | 22 | 0.44221 | 1 | 0.36219 | 0.44106 | 1 | 0.87804 | 0.04 |
| Lysine degradation | 30 | 0.60302 | 1 | 0.45927 | 0.33793 | 1 | 1 | 0.03571 |
| Inositol phosphate metabolism | 30 | 0.60302 | 1 | 0.45927 | 0.33793 | 1 | 1 | 0.05 |
| Cysteine and methionine metabolism | 33 | 0.66332 | 1 | 0.49185 | 0.30817 | 1 | 1 | 0 |
| Glycerophospholipid metabolism | 36 | 0.72362 | 1 | 0.52252 | 0.2819 | 1 | 1 | 0.03774 |
| Purine metabolism | 70 | 1.407 | 1 | 0.76623 | 0.11564 | 1 | 1 | 0.01124 |

**Table S13**. Pathway enrichment output generated by MetaboAnalyst for AuTM treatment in A2780 cells.

|  | Total | Expected | Hits | p-value | log10(p-value) | Holm adjust | FDR | Impact |
| --- | --- | --- | --- | --- | --- | --- | --- | --- |
| Alanine, aspartate and glutamate metabolism | 28 | 0.33417 | 7 | 1.02E-08 | 7.9906 | 8.17E-07 | 8.17E-07 | 0.21875 |
| Glyoxylate and dicarboxylate metabolism | 32 | 0.38191 | 5 | 2.26E-05 | 4.6452 | 0.001788 | 0.000905 | 0.13333 |
| Citrate cycle (TCA cycle) | 20 | 0.23869 | 4 | 6.24E-05 | 4.205 | 0.004865 | 0.001557 | 0.17242 |
| Valine, leucine and isoleucine biosynthesis | 8 | 0.095477 | 3 | 7.78E-05 | 4.1088 | 0.005994 | 0.001557 | 0.25 |
| Glycolysis or Gluconeogenesis | 26 | 0.3103 | 4 | 0.000184 | 3.7355 | 0.013973 | 0.002942 | 0.14705 |
| Arginine and proline metabolism | 36 | 0.42965 | 4 | 0.000671 | 3.1731 | 0.050342 | 0.00895 | 0.07896 |
| Phenylalanine, tyrosine and tryptophan biosynthesis | 4 | 0.047739 | 2 | 0.000799 | 3.0976 | 0.059099 | 0.009127 | 0.75 |
| Pyruvate metabolism | 23 | 0.2745 | 3 | 0.002197 | 2.6582 | 0.16039 | 0.021971 | 0.10714 |
| Phenylalanine metabolism | 8 | 0.095477 | 2 | 0.003622 | 2.441 | 0.26079 | 0.032196 | 0.375 |
| Glycine, serine and threonine metabolism | 33 | 0.39384 | 3 | 0.006275 | 2.2024 | 0.44551 | 0.050199 | 0.21951 |
| Valine, leucine and isoleucine degradation | 40 | 0.47739 | 3 | 0.010776 | 1.9675 | 0.75433 | 0.073839 | 0.05661 |
| Arginine biosynthesis | 14 | 0.16709 | 2 | 0.011279 | 1.9477 | 0.77824 | 0.073839 | 0.11765 |
| Tyrosine metabolism | 42 | 0.50126 | 3 | 0.012333 | 1.9089 | 0.83865 | 0.073839 | 0.11628 |
| Butanoate metabolism | 15 | 0.17902 | 2 | 0.012922 | 1.8887 | 0.86576 | 0.073839 | 0.06667 |
| Glutathione metabolism | 28 | 0.33417 | 2 | 0.042422 | 1.3724 | 1 | 0.21211 | 0.05406 |
| Lipoic acid metabolism | 28 | 0.33417 | 2 | 0.042422 | 1.3724 | 1 | 0.21211 | 0.07317 |
| Porphyrin metabolism | 31 | 0.36997 | 2 | 0.051091 | 1.2917 | 1 | 0.24043 | 0.07408 |
| Nitrogen metabolism | 6 | 0.071608 | 1 | 0.069611 | 1.1573 | 1 | 0.30938 | 0.25 |
| Taurine and hypotaurine metabolism | 8 | 0.095477 | 1 | 0.091777 | 1.0373 | 1 | 0.38643 | 0.28571 |
| Primary bile acid biosynthesis | 46 | 0.54899 | 2 | 0.10234 | 0.98996 | 1 | 0.40935 | 0.08164 |
| Histidine metabolism | 16 | 0.19095 | 1 | 0.17553 | 0.75564 | 1 | 0.6687 | 0.06667 |
| Ubiquinone and other terpenoid-quinone biosynthesis | 19 | 0.22676 | 1 | 0.20501 | 0.68822 | 1 | 0.68677 | 0.0625 |
| Fructose and mannose metabolism | 20 | 0.23869 | 1 | 0.21462 | 0.66834 | 1 | 0.68677 | 0 |
| Selenocompound metabolism | 20 | 0.23869 | 1 | 0.21462 | 0.66834 | 1 | 0.68677 | 0 |
| Pantothenate and CoA biosynthesis | 20 | 0.23869 | 1 | 0.21462 | 0.66834 | 1 | 0.68677 | 0.05263 |
| Propanoate metabolism | 22 | 0.26256 | 1 | 0.23349 | 0.63173 | 1 | 0.71844 | 0.04 |
| One carbon pool by folate | 26 | 0.3103 | 1 | 0.26996 | 0.5687 | 1 | 0.79663 | 0.09259 |
| Galactose metabolism | 27 | 0.32224 | 1 | 0.27882 | 0.55467 | 1 | 0.79663 | 0.03125 |
| Cysteine and methionine metabolism | 33 | 0.39384 | 1 | 0.32986 | 0.48166 | 1 | 0.90997 | 0 |
| Amino sugar and nucleotide sugar metabolism | 42 | 0.50126 | 1 | 0.40006 | 0.39788 | 1 | 1 | 0.02326 |

**Table S14**. Pathway enrichment output generated by MetaboAnalyst for CN1 treatment in A2780 cells.

|  | Total | Expected | Hits | p-value | log10(p-value) | Holm adjust | FDR | Impact |
| --- | --- | --- | --- | --- | --- | --- | --- | --- |
| Glycolysis or Gluconeogenesis | 26 | 0.42462 | 6 | 1.90E-06 | 5.7206 | 0.000152 | 0.0001 | 0.20587 |
| Alanine, aspartate and glutamate metabolism | 28 | 0.45729 | 6 | 3.05E-06 | 5.5162 | 0.000241 | 0.0001 | 0.34375 |
| Valine, leucine and isoleucine biosynthesis | 8 | 0.13065 | 4 | 3.75E-06 | 5.4255 | 0.000293 | 0.0001 | 0.5 |
| Glyoxylate and dicarboxylate metabolism | 32 | 0.52261 | 5 | 0.000116 | 3.9361 | 0.008921 | 0.002317 | 0.13333 |
| Phenylalanine, tyrosine and tryptophan biosynthesis | 4 | 0.065327 | 2 | 0.001509 | 2.8213 | 0.11468 | 0.024143 | 0.75 |
| Valine, leucine and isoleucine degradation | 40 | 0.65327 | 4 | 0.00343 | 2.4647 | 0.25723 | 0.041079 | 0.07548 |
| Nitrogen metabolism | 6 | 0.09799 | 2 | 0.003697 | 2.4322 | 0.27357 | 0.041079 | 0.25 |
| Amino sugar and nucleotide sugar metabolism | 42 | 0.68593 | 4 | 0.004108 | 2.3864 | 0.29988 | 0.041079 | 0.09303 |
| Pyruvate metabolism | 23 | 0.37563 | 3 | 0.005518 | 2.2583 | 0.39727 | 0.049045 | 0.14285 |
| Phenylalanine metabolism | 8 | 0.13065 | 2 | 0.006763 | 2.1698 | 0.48019 | 0.054106 | 0.375 |
| Galactose metabolism | 27 | 0.44095 | 3 | 0.008726 | 2.0592 | 0.6108 | 0.06346 | 0.09375 |
| Glycine, serine and threonine metabolism | 33 | 0.53894 | 3 | 0.015252 | 1.8167 | 1 | 0.10168 | 0.2439 |
| Arginine and proline metabolism | 36 | 0.58794 | 3 | 0.019321 | 1.714 | 1 | 0.11826 | 0.05264 |
| Arginine biosynthesis | 14 | 0.22864 | 2 | 0.020695 | 1.6841 | 1 | 0.11826 | 0.2353 |
| Butanoate metabolism | 15 | 0.24497 | 2 | 0.023641 | 1.6263 | 1 | 0.12609 | 0.06667 |
| Citrate cycle (TCA cycle) | 20 | 0.32663 | 2 | 0.040696 | 1.3905 | 1 | 0.20348 | 0.13794 |
| One carbon pool by folate | 26 | 0.42462 | 2 | 0.065582 | 1.1832 | 1 | 0.30862 | 0.11111 |
| Glutathione metabolism | 28 | 0.45729 | 2 | 0.074782 | 1.1262 | 1 | 0.31487 | 0.05406 |
| Lipoic acid metabolism | 28 | 0.45729 | 2 | 0.074782 | 1.1262 | 1 | 0.31487 | 0.07317 |
| Porphyrin metabolism | 31 | 0.50628 | 2 | 0.089308 | 1.0491 | 1 | 0.35723 | 0.07408 |
| Taurine and hypotaurine metabolism | 8 | 0.13065 | 1 | 0.12368 | 0.9077 | 1 | 0.47116 | 0.28571 |
| Pyrimidine metabolism | 39 | 0.63693 | 2 | 0.13156 | 0.88087 | 1 | 0.4784 | 0.05263 |
| Ascorbate and aldarate metabolism | 9 | 0.14698 | 1 | 0.13806 | 0.85992 | 1 | 0.48023 | 0.125 |
| Tyrosine metabolism | 42 | 0.68593 | 2 | 0.14845 | 0.82842 | 1 | 0.48711 | 0.11628 |
| Biotin metabolism | 10 | 0.16332 | 1 | 0.15222 | 0.81752 | 1 | 0.48711 | 0 |
| Primary bile acid biosynthesis | 46 | 0.75126 | 2 | 0.17164 | 0.76539 | 1 | 0.52811 | 0.08164 |
| Histidine metabolism | 16 | 0.26131 | 1 | 0.23258 | 0.63342 | 1 | 0.68371 | 0.06667 |
| Starch and sucrose metabolism | 18 | 0.29397 | 1 | 0.2577 | 0.58888 | 1 | 0.68371 | 0.12 |
| Ubiquinone and other terpenoid-quinone biosynthesis | 19 | 0.3103 | 1 | 0.26996 | 0.5687 | 1 | 0.68371 | 0.0625 |
| Pentose and glucuronate interconversions | 19 | 0.3103 | 1 | 0.26996 | 0.5687 | 1 | 0.68371 | 0.06667 |
| Fructose and mannose metabolism | 20 | 0.32663 | 1 | 0.28203 | 0.5497 | 1 | 0.68371 | 0 |
| Selenocompound metabolism | 20 | 0.32663 | 1 | 0.28203 | 0.5497 | 1 | 0.68371 | 0 |
| Pantothenate and CoA biosynthesis | 20 | 0.32663 | 1 | 0.28203 | 0.5497 | 1 | 0.68371 | 0.05263 |
| Propanoate metabolism | 22 | 0.3593 | 1 | 0.30559 | 0.51486 | 1 | 0.71904 | 0.04 |
| Lysine degradation | 30 | 0.48995 | 1 | 0.39261 | 0.40604 | 1 | 0.87247 | 0.03571 |
| Inositol phosphate metabolism | 30 | 0.48995 | 1 | 0.39261 | 0.40604 | 1 | 0.87247 | 0.05 |
| Cysteine and methionine metabolism | 33 | 0.53894 | 1 | 0.42246 | 0.37421 | 1 | 0.91343 | 0 |
| Glycerophospholipid metabolism | 36 | 0.58794 | 1 | 0.45089 | 0.34593 | 1 | 0.94925 | 0.03774 |
| Purine metabolism | 70 | 1.1432 | 1 | 0.69229 | 0.15971 | 1 | 1 | 0.01124 |

**Table S15**. Pathway enrichment output generated by MetaboAnalyst for CN2 treatment in A2780 cells.

|  | Total | Expected | Hits | p-value | log10(p-value) | Holm adjust | FDR | Impact |
| --- | --- | --- | --- | --- | --- | --- | --- | --- |
| Glyoxylate and dicarboxylate metabolism | 32 | 0.62312 | 8 | 6.03E-08 | 7.2195 | 4.83E-06 | 4.83E-06 | 0.2 |
| Alanine, aspartate and glutamate metabolism | 28 | 0.54523 | 7 | 4.63E-07 | 6.3342 | 3.66E-05 | 1.85E-05 | 0.375 |
| Glycolysis or Gluconeogenesis | 26 | 0.50628 | 6 | 5.76E-06 | 5.2395 | 0.000449 | 0.000154 | 0.20587 |
| Valine, leucine and isoleucine biosynthesis | 8 | 0.15578 | 4 | 7.82E-06 | 5.1068 | 0.000602 | 0.000156 | 0.5 |
| One carbon pool by folate | 26 | 0.50628 | 4 | 0.001306 | 2.8842 | 0.099221 | 0.020889 | 0.14815 |
| Glutathione metabolism | 28 | 0.54523 | 4 | 0.00174 | 2.7595 | 0.13048 | 0.023197 | 0.24325 |
| Phenylalanine, tyrosine and tryptophan biosynthesis | 4 | 0.077889 | 2 | 0.00215 | 2.6676 | 0.15909 | 0.024569 | 0.75 |
| Glycine, serine and threonine metabolism | 33 | 0.64259 | 4 | 0.003247 | 2.4885 | 0.23706 | 0.032474 | 0.39024 |
| Nitrogen metabolism | 6 | 0.11683 | 2 | 0.005245 | 2.2803 | 0.37764 | 0.046622 | 0.25 |
| Citrate cycle (TCA cycle) | 20 | 0.38945 | 3 | 0.006094 | 2.2151 | 0.43265 | 0.047939 | 0.17242 |
| Valine, leucine and isoleucine degradation | 40 | 0.77889 | 4 | 0.006592 | 2.181 | 0.46142 | 0.047939 | 0.07548 |
| Amino sugar and nucleotide sugar metabolism | 42 | 0.81784 | 4 | 0.007856 | 2.1048 | 0.54205 | 0.052372 | 0.09303 |
| Pyruvate metabolism | 23 | 0.44786 | 3 | 0.009099 | 2.041 | 0.61873 | 0.054601 | 0.14285 |
| Phenylalanine metabolism | 8 | 0.15578 | 2 | 0.009555 | 2.0198 | 0.64019 | 0.054601 | 0.375 |
| Galactose metabolism | 27 | 0.52575 | 3 | 0.014256 | 1.846 | 0.94092 | 0.076034 | 0.09375 |
| Cysteine and methionine metabolism | 33 | 0.64259 | 3 | 0.024576 | 1.6095 | 1 | 0.12288 | 0.09091 |
| Arginine biosynthesis | 14 | 0.27261 | 2 | 0.028876 | 1.5395 | 1 | 0.13589 | 0.2353 |
| Butanoate metabolism | 15 | 0.29209 | 2 | 0.032919 | 1.4826 | 1 | 0.14631 | 0.06667 |
| Histidine metabolism | 16 | 0.31156 | 2 | 0.037171 | 1.4298 | 1 | 0.15651 | 0.26667 |
| Lipoic acid metabolism | 28 | 0.54523 | 2 | 0.10143 | 0.99383 | 1 | 0.40573 | 0.07317 |
| Porphyrin metabolism | 31 | 0.60364 | 2 | 0.12042 | 0.9193 | 1 | 0.45874 | 0.07408 |
| Arginine and proline metabolism | 36 | 0.70101 | 2 | 0.15381 | 0.81301 | 1 | 0.55931 | 0.02632 |
| Ascorbate and aldarate metabolism | 9 | 0.17525 | 1 | 0.16258 | 0.78893 | 1 | 0.5655 | 0.125 |
| Pyrimidine metabolism | 39 | 0.75942 | 2 | 0.17466 | 0.75781 | 1 | 0.57274 | 0.05263 |
| Biotin metabolism | 10 | 0.19472 | 1 | 0.17898 | 0.74719 | 1 | 0.57274 | 0 |
| Tyrosine metabolism | 42 | 0.81784 | 2 | 0.19596 | 0.70784 | 1 | 0.60295 | 0.11628 |
| D-Amino acid metabolism | 15 | 0.29209 | 1 | 0.25642 | 0.59104 | 1 | 0.75978 | 0.125 |
| Starch and sucrose metabolism | 18 | 0.3505 | 1 | 0.29945 | 0.52368 | 1 | 0.79219 | 0.12 |
| Ubiquinone and other terpenoid-quinone biosynthesis | 19 | 0.36997 | 1 | 0.31324 | 0.50412 | 1 | 0.79219 | 0.0625 |
| Pentose and glucuronate interconversions | 19 | 0.36997 | 1 | 0.31324 | 0.50412 | 1 | 0.79219 | 0.06667 |
| Fructose and mannose metabolism | 20 | 0.38945 | 1 | 0.32678 | 0.48575 | 1 | 0.79219 | 0 |
| Selenocompound metabolism | 20 | 0.38945 | 1 | 0.32678 | 0.48575 | 1 | 0.79219 | 0 |
| Pantothenate and CoA biosynthesis | 20 | 0.38945 | 1 | 0.32678 | 0.48575 | 1 | 0.79219 | 0.05263 |
| beta-Alanine metabolism | 21 | 0.40892 | 1 | 0.34005 | 0.46845 | 1 | 0.80012 | 0 |
| Propanoate metabolism | 22 | 0.42839 | 1 | 0.35308 | 0.45213 | 1 | 0.80703 | 0.04 |
| Lysine degradation | 30 | 0.58417 | 1 | 0.44867 | 0.34807 | 1 | 0.97011 | 0.03571 |
| Inositol phosphate metabolism | 30 | 0.58417 | 1 | 0.44867 | 0.34807 | 1 | 0.97011 | 0.05 |
| Sphingolipid metabolism | 32 | 0.62312 | 1 | 0.47035 | 0.32758 | 1 | 0.9902 | 0.02174 |
| Glycerophospholipid metabolism | 36 | 0.70101 | 1 | 0.51125 | 0.29137 | 1 | 1 | 0.03774 |
| Primary bile acid biosynthesis | 46 | 0.89573 | 1 | 0.60058 | 0.22143 | 1 | 1 | 0.04082 |
| Purine metabolism | 70 | 1.3631 | 1 | 0.75526 | 0.1219 | 1 | 1 | 0.01124 |

**Table S16**. List of metabolic pathways (KEGG) inferred from the metabolites identified in the ^1^H NMR spectra of A2780 and SKOV-3 cells. The number of metabolites detected for each pathway (hits) is also reported.

| metabolic pathway | Hits A2780 | Hits SKOV-3 |
| --- | --- | --- |
| Alanine, aspartate and glutamate metabolism | 9 | 6 |
| Amino sugar and nucleotide sugar metabolism | 4 | 1 |
| Arginine and proline metabolism | 7 | 4 |
| Arginine biosynthesis | 6 | 3 |
| Ascorbate and aldarate metabolism | 1 | 1 |
| beta-Alanine metabolism | 2 | 2 |
| Biotin metabolism | 1 | 1 |
| Butanoate metabolism | 2 | 2 |
| Citrate cycle (TCA cycle) | 4 | 4 |
| Cysteine and methionine metabolism | 3 | 3 |
| D-Amino acid metabolism | 1 | 1 |
| Ether lipid metabolism | 1 | 1 |
| Fructose and mannose metabolism | 1 | 1 |
| Galactose metabolism | 3 | 2 |
| Glutathione metabolism | 5 | 4 |
| Glycerophospholipid metabolism | 3 | 3 |
| Glycine, serine and threonine metabolism | 5 | 6 |
| Glycolysis | 6 | 5 |
| Glyoxylate and dicarboxylate metabolism | 8 | 7 |
| Histidine metabolism | 3 | 2 |
| Inositol phosphate metabolism | 1 | 1 |
| Lipoic acid metabolism | 2 | 2 |
| Lysine degradation | 1 | 1 |
| Nicotinate and nicotinamide metabolism | 2 | 2 |
| Nitrogen metabolism | 2 | 1 |
| One carbon pool by folate | 5 | 5 |
| Pantothenate and CoA biosynthesis | 2 | 2 |
| Pentose and glucuronate interconversions | 1 | 0 |
| Phenylalanine metabolism | 2 | 2 |
| Porphyrin metabolism | 2 | 2 |
| Propanoate metabolism | 1 | 1 |
| Purine metabolism | 7 | 5 |
| Pyrimidine metabolism | 1 | 2 |
| Pyruvate metabolism | 4 | 4 |
| Selenocompound metabolism | 1 | 1 |
| Sphingolipid metabolism | 1 | 1 |
| Taurine and hypotaurine metabolism | 1 | 0 |
| Tryptophan metabolism | 0 | 1 |
| Tyrosine metabolism | 3 | 3 |
| Ubiquinone and other terpenoid-quinone biosynthesis | 1 | 1 |
| Valine, leucine and isoleucine degradation | 3 | 3 |

**Table S17**. Concentrations (mg/L) of representative nutrients in the cell culture media at two time points: fresh medium and after 24 h of cell growth. For each metabolite, the corresponding cellular uptake, calculated as the difference between the initial and 24 h concentrations, is also reported.

|  | A2780  mg/L | | | SKOV-3  mg/L | | |
| --- | --- | --- | --- | --- | --- | --- |
|  | RPMI 1640 | 24 h | Taken up | DMEM | 24 h | Taken up |
| Val | 20 | 6.8 | 13.2 | 93.6 | 85.8 | 7.7 |
| Leu | 50 | 23.9 | 26.1 | 104.8 | 89.0 | 15.8 |
| Ile | 50 | 24.5 | 25.5 | 104.8 | 87.6 | 17.2 |
|  |  |  |  |  |  |  |
| Phe | 15 | 11.2 | 3.8 | 66 | 68.0 | 0 |
| Tyr | 28.8 | 13.7 | 15.1 | 72 | 68.3 | 3.7 |
| His | 15 | 8.1 | 6.9 | 42 | 39.3 | 2.7 |
|  |  |  |  |  |  |  |
| Glucose | 2000 | 847.8 | 1152.2 | 4500 | 4070.5 | 429.5 |

**Table S18**. Cluster analysis based on cell lines of PCA score plot derived from Bspectra of treated cell lysates. Centroid Euclidean distance matrix.

|  | A2780 | SKOV-3 |
| --- | --- | --- |
| A2780 | 0 | 4.633 |
| SKOV-3 | 4.633 | 0 |

**Table S19**. Cluster analysis based on cell lines of PCA score plot derived from Bspectra of treated growth media. Centroid Euclidean distance matrix.

|  | A2780 | SKOV-3 |
| --- | --- | --- |
| A2780 | 0 | 14.246 |
| SKOV-3 | 14.246 | 0 |

**Table S20**. Cluster analysis based on treatments of PCA score plot derived from Bspectra of treated cell lysates. Centroid Euclidean distance matrix.

|  | AF | AuTM | CN1\CN2 |
| --- | --- | --- | --- |
| AF | 0 | 0.581 | 2.243 |
| AuTM | 0.581 | 0 | 1.762 |
| CN1\CN2 | 2.243 | 1.762 | 0 |

**Table S21**. Cluster analysis based on treatments of PCA score plot derived from Bspectra of treated cell lysates. Centroid Euclidean distance matrix.

|  | AF | AuTM | CN1\CN2 |
| --- | --- | --- | --- |
| AF | 0 | 2.033 | 4.880 |
| AuTM | 2.033 | 0 | 6.871 |
| CN1\CN2 | 4.880 | 6.871 | 0 |

**Table S22**. Statistical validation and quality parameters for supervised (PLS-DA) multivariate models. The parameters include the number of predictive components, cumulative explained variance for the data matrix (R^2^X(cum)) and class assignment (R^2^Y (cum)), and the cumulative cross-validated predictability (Q^2^ (cum)). RMSEE indicates the Root Mean Square Error of Estimation. p(R^2^Y) and p(Q^2^) values represent the probability of the real model performing similarly to random permutations. Cum= cumulative.

| Model Matrix | Components (predictive) | R^2^X (cum) | R^2^Y (cum) | Q^2^ (cum) | RMSEE | Permutation  p(R^2^Y) | Permutation p(Q^2^) |
| --- | --- | --- | --- | --- | --- | --- | --- |
| Cell  Lysates | 2 | 0.680 | 0.140 | 0.039 | 0.384 | 0.010 | 0.005 |
| Growth  Media | 2 | 0.666 | 0.150 | 0.036 | 0.383 | 0.025 | 0.010 |

**Table S23**. Cluster analysis based on cell lines of PCA score plot derived from concatenated average Bdifference_spectra (cell lysates + growth media). Centroid Euclidean distance matrix.

|  | A2780 | SKOV-3 |
| --- | --- | --- |
| A2780 | 0 | 1.845 |
| SKOV-3 | 1.845 | 0 |

**Table S24**. Cluster analysis based on treatments of PCA score plot derived from concatenated average Bdifference_spectra (cell lysates + growth media). Centroid Euclidean distance matrix.

|  | AF | AuTM | CN1\CN2 |
| --- | --- | --- | --- |
| AF | 0 | 1.859 | 6.831 |
| AuTM | 1.859 | 0 | 5.649 |
| CN1\CN2 | 6.831 | 5.649 | 0 |
